# Semi-Analytical Approach and Green’s Function Method: A Comparison in the Analysis of the Interaction of a Moving Mass on an Infinite Beam on a Three-Layer Viscoelastic Foundation at the Stability Limit—The Effect of Damping of Foundation Materials

**DOI:** 10.3390/ma17020279

**Published:** 2024-01-05

**Authors:** Zuzana Dimitrovová, Traian Mazilu

**Affiliations:** 1 Department of Civil Engineering, NOVA School of Science and Technology, NOVA University of Lisbon, 2829-516 Caparica, Portugal; zdim@fct.unl.pt; 2 IDMEC, Instituto Superior Técnico, Universidade de Lisboa, 1049-001 Lisbon, Portugal; 3 Department of Railway Vehicles, Faculty of Transport, National University of Science and Technology Politehnica Bucharest, 060042 Bucharest, Romania

**Keywords:** Green’s function method, semi-analytical approach, integral transforms, moving mass instability, railway track

## Abstract

In this paper, the interaction of a mass moving uniformly on an infinite beam on a three-layer viscoelastic foundation is analyzed with the objective of determining the lowest velocity at the stability limit, called, in this context, the critical velocity. This issue is important for rail transport and, in particular, for the high-speed train, because the moving mass is the basic model of a vehicle, and the infinite beam on a three-layer viscoelastic foundation is the usual mechanical representation of the railway track. In addition to this, the advantages and disadvantages of the two implemented methods, namely, the semi-analytical approach and the Green’s function method, are summarized in terms of computational time, the precision of the obtained results, limitations, and the feasibility of implementation. All results are presented in a dimensionless form to cover a wide range of possible scenarios. Some results may be considered academic, however, results related to a particular railway track are also included. Particular attention is paid to the influence of the damping of materials in the foundation upon the critical velocity of the moving mass. Regarding the semi-analytical approach, it is demonstrated that the critical velocities can be obtained in an exact manner by tracing the branches of the so-called instability lines in the velocity–moving-mass plane. This analysis can be maintained within the real domain. As for the time series, they can be determined by a numerical inverse Laplace transform. Moreover, thanks to the analytical form of the final result in the Fourier domain, each value corresponding to a specific time instant can be obtained directly, that is, without the previous time history. Regarding the Green’s function method, this is used to verify a few points delimiting the stable and unstable regions of the moving mass with the help of the D-decomposition approach. Additionally, a numerical algorithm based on the Green’s function and convolution integral written for dimensionless quantities is used to calculate the time series of the moving mass. In addition to identifying the critical velocity of the moving mass, its connection with the critical velocity of the moving force is emphasized, and the possibility of validating the results on long finite beams using modal expansion is presented and described.

## 1. Introduction

The railway has the function of providing for the circulation of railway vehicles in a safe, economical, and comfortable way. For all of these aspects to be guaranteed, it is necessary that there is an adequate transfer of efforts to the ground, taking into account the high demands that the rolling stock imposes on the rail; therefore, one of the most challenging problems in the development of railway passenger transport is the identification of the most suitable technical solutions capable of ensuring an increase in the traffic speed under standardized conditions with regard to the comfort of passengers and the ride quality of the vehicles. From this point of view, the quality of the track plays a determining role in terms of the intensity of mechanical stresses to which the entire vehicle/track system is exposed during operation. Indeed, many reports have indicated a sudden increase in track deformation and acceleration at the level of the rolling gear of the vehicle associated with exceeding a certain speed threshold when trains run on excessively flexible tracks [1,2]. The starting point for overcoming such obstacles is a better knowledge of the nature of the interaction phenomena between the vehicle and the track based on theoretical and experimental approaches. 

**Figure 1:**
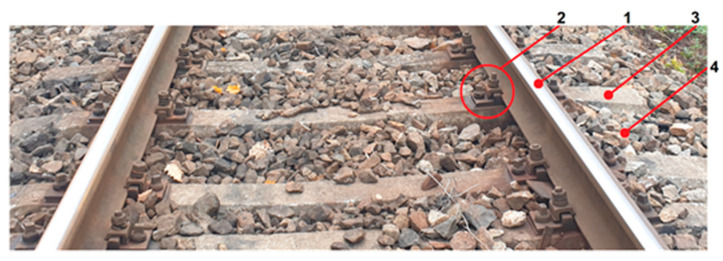
Track structure: 1. rail; 2. rail fastening system; 3. concrete sleeper; 4. ballast.

Mechanical representations of the track must consider its structure and its static and dynamic properties associated with the frequency domain of interest. In the following discussion, the structure of the classic track with concrete sleepers is considered. Figure 1 shows the main components of a classic track. The two rails of the track are placed on concrete sleepers, separated by rail pads, and clamped to the sleepers by the rail fastening system, which includes the sleeper bolt and a tension clamp. The frame, consisting of the two rails and the concrete sleepers, is placed on the crushed stone prism (ballast). The elasticity and damping of the track are mainly ensured by the materials from which the rail pads, ballast, and subgrade (the layer under the ballast) are made.

In the range of very low frequencies (0.5–20 Hz), that is, in the range in which the natural frequencies of the vehicle carbody and its suspensions are located, the track model can be composed of bodies with concentrated parameters [3,4,5] or even be rigid [6,7]. This is because, on the one hand, the receptance of the vehicle train at the wheels is much higher than the track receptance in this frequency range, and due to this, the wheels adopt the displacement caused by the track’s irregularities, while the rail remains practically fixed. On the other hand, the bending waves of the rail are evanescent, so the coupling between vehicle wheels due to this type of wave can be neglected. 

At low and medium frequencies (6–700 Hz), the track vibrates like a two-DOF oscillator, exhibiting two natural frequencies and an antiresonance frequency between them. In addition to evanescent waves, there are also propagating waves that arise between the low natural frequency and the antiresonance frequency and at frequencies higher than the high natural frequency [8]. The track model is based on continuous medium theory, in which the rail is modeled as an Euler–Bernoulli or Timoshenko beam, the sleepers are assimilated by a purely inertial layer, and the viscoelastic properties of the rail pads and ballast prism are modeled using the Winkler foundation with damping, which comprises the so-called two-layer model. Additional shear effects can be introduced considering a two-parameter (i.e., Pasternak) foundation [9,10]. However, it would be more realistic to include dynamically activated mass in the foundation as well [11]. A characteristic feature of this frequency range is the fact that the rail response does not change significantly along the sleeper spacing, which justifies neglecting the influence of equidistantly spaced sleepers on the rail receptance. 

When the frequency increases, the response of the rail changes dramatically due to the influence of the bending modes of the rail on the sleepers. Among them, the most influential is the first eigenmode, whose wavelength is equal to twice the distance between the sleepers. The rail receptance shows a maximum at the mid-span between the sleepers at the pinned–pinned resonance frequency and a minimum (antiresonance) above the sleepers but at a slightly higher frequency. To simulate this rail behavior, the track model should consist of a beam resting on discrete supports [12,13,14].

Several discretization methods for the continuous elastic medium can be used to improve the accuracy of the prediction of the dynamic regime of the track. Thus, for problems specific to high frequencies, the finite element method is used because it allows a more accurate mechanical description of the track components [15,16,17]. Particularly complex behavior of the components in a ballast bed can be studied using the discrete element method [18,19]. To solve problems related to running noise, the boundary element method is used [20,21]. In this context, the moving element method [22,23] and the new moving beam element [24] or the novel model of nonlinear dynamic interaction [25] should also be mentioned.

The track representation is useful for investigating many of the issues that the vehicle/track interaction raises for railway engineers and researchers. An interesting topic from a technical and academic point of view is the phenomenon of the instability of the vehicle/track system, which can be observed in certain cases when the velocity of the vehicle exceeds the velocity of propagation of the elastic waves generated in the track. This occurs when the track infrastructure is built on poorly consistent soils [1,2,26] and/or the rails are subject to excessive expansion [27,28]. Indeed, when the velocity of a moving object on a continuous elastic structure exceeds the velocity of propagation of the elastic waves induced in the structure, the moving object radiates anomalous Doppler waves that produce a negative damping effect at the object/structure interface, resulting in the destabilization of the whole system under certain circumstances [29,30]. And this phenomenon could occur in the case of a railway vehicle if the above conditions are met.

The problem of the stability of a moving object on an elastic structure with applications to the vehicle/track system was treated in the past by considering different mechanical representations of the vehicle (moving object) and the track (elastic structure). A railway vehicle can be represented by simple models, such as a moving mass [28,31,32,33], two moving masses [34,35], a two-mass oscillator [36,37,38] or a train of two-mass oscillators [39,40,41], a three-mass oscillator [42], or a three-body oscillator [43,44]. The set of moving inertial objects was also dealt with in pioneering works [45,46] and, more recently, in [47]. A more complex vehicle model can also be used to study the stability issue [48,49]. The track is modeled using an Euler–Bernoulli infinite beam on a continuous foundation with one elastic layer [28,29,31,32,34] or two [35,50,51] or three elastic layers [52]. Instead of the Euler–Bernoulli beam, the Timoshenko beam can also be used [42,43,53]. In order to take into account the influence of the sleeper bay, a model of an infinite beam on a foundation with discrete supports should be considered for the track [54]. 

Other works concern the limitations of classical beam theories [55,56,57]. Large deformations are investigated in [58], initial conditions are accounted for in [59], and the non-uniform motion of the inertial object is analyzed in [60]. Related problems include the variation in foundation stiffness [61,62] or analyses concerned with tracks other than classical ballasted tracks [63]. Related problems are also of interest, for instance, complex issues in which a moving object interacts with an elastic structure, as happens in a pantograph–catenary system [64,65].

The main objective of the analyses presented in this paper is to identify the critical velocity of the moving object, which, in this context, will indicate the lowest velocity at which the moving object/elastic structure system becomes unstable. In this regard, it is necessary to emphasize that the critical velocity of the moving force, i.e., of an object without inertia, is different and not related to the anomalous Doppler effect. Nevertheless, the critical velocity of the moving force is closely related to the aim of this paper. This topic has been deeply analyzed over the course of several years: pioneering works are summarized in the monograph in [66], more complicated models are approached analytically in [67], and more recently, artificial intelligence has also been used for this complicated problem in real situations [68].

When investigating the quality of a system to be stable according to the velocity of a moving object, in certain cases, depending on the nature of the moving object and the elastic structure, zones of stability alternating with zones of instability have been identified [42,51,52], but the first zone on the velocity axis is always a zone of stability, and therefore, the critical velocity of a moving object refers to the transition from this zone to the first zone of instability that follows it.

The determination of stability/instability zones and critical velocity is related to the nature of the roots of the characteristic equation associated with the linear equations of motion of the moving object/elastic structure system. All roots of the characteristic equation have a negative real part in the stability zone, and at least one solution of the characteristic equation has a positive real part in the instability zone. The difficulty of the problem lies in the fact that the characteristic equation does not have a polynomial form, and therefore, the solution to the problem can be obtained by a graph-analytical method, the so-called D-decomposition method [69]. This method maps the zones where the characteristic equation has stable or unstable roots for a given value of the moving object velocity in the complex plane associated with the parameter of interest of the moving object/elastic structure system by sweeping the angular frequency axis [26,28,29,36,42,43,44,48,50,70].

Another objective of the problem of the stability of a moving object on an elastic structure refers to the description of the dynamic behavior when the system loses its stability to assess the potential consequences. Achieving this goal requires taking into account the loss of contact between the moving object and the structure. In this case, the equations of motion become nonlinear, and solving them in the time domain can be performed using a method based on the Green’s function [32,40]. In this way, a nonlinear stability analysis based on the Hopf bifurcation is possible [42]. 

Relatively recently, a new semi-analytical method for analyzing the response of an elastic structure to a moving object has been proposed [10,11,34,35,38,51,52,59]. The main objective of this method is to determine the full dynamic response of the structure directly as a sum of residues. In this way, the vibrations of the structure can be monitored from the initial instant with respect to inhomogeneous initial conditions [59] and without loss of accuracy. Very good results are achieved with one-layer and two-layer models [10,34,35,38]. An indication of the stability of the moving object is then obtained as a secondary result with respect to the main objective mentioned above. For a more complex supporting structure, the sum of residues must be supplemented by additional numerical integration [11] due to discontinuities in the equivalent flexibility of the supporting structure. However, these discontinuities never occur in unstable regions; therefore, stability or instability can also be identified directly from the characteristic equation, as in the D-decomposition method. In the solution method, the variable related to the frequency is modified so that unstable cases are identified by the existence of at least one root with a negative imaginary part. The identification of instability starts with the selection of the velocity of the moving object, and for such a value, a real-valued frequency providing a real-valued equivalent flexibility is found using the classic intersection method, and then the corresponding moving mass is calculated as a real-valued result. Each time the equivalent flexibility is required, it is precisely calculated by contour integration, which ensures the high accuracy of the obtained results. A smooth change in velocity will cause a smooth change in the value of the moving mass. As a result, the so-called instability lines, which can have several branches, are obtained. After scanning the whole velocity–moving-mass plane, by choosing a particular value for the moving mass, velocity intervals of stability and instability can be obtained from the intersection with instability branches. These intervals also indicate the number of unstable components contained in the full solution [52].

With regard to the previous discussion, it can be concluded that the numerical evaluation of the dynamic behavior of railways or, in general, structures subjected to moving loads is undergoing great development, both with the use of numerical methods and with the help of other approaches, such as semi-analytical methods or methods implementing the dynamic Green’s function.

This paper is focused on an infinite beam supported by three viscoelastic layers, which, due to its computational efficiency and relatively good approximation to reality, is commonly used by railway engineers for the evaluation of several relevant aspects. The new findings that are presented concern the instability of a moving mass. The critical velocity in this context will be used for the lowest velocity that separates stable and unstable behavior. However, the new contribution of this paper lies not only in the presentation of relevant results but also in the detailed comparison of the semi-analytical and dynamic Green’s function approach from the viewpoint of several aspects, such as computational efficiency, the accuracy of the obtained results, ease of implementation, limitations, etc.

It is demonstrated that there are several situations with rather unpredictable behavior, especially at low damping levels, as already shown for simpler, one-layer [34] and two-layer models [35,51]. In the semi-analytical approach, the so-called instability lines are traced, indicating the moving mass as a function of velocity in a limiting situation where one of the induced frequencies is real-valued. It is shown that these lines have several branches. They can have quite a strange development, as they can form a closed curve. Open curves must end with asymptotes tending to infinite mass at a fixed velocity, except for at least one end of an instability branch, which tends to zero mass at infinite velocity. 

It is also shown that the expected relation to the critical velocity of the moving force is not confirmed in all cases. First, three values of such a critical velocity do not always exist. Sometimes, there is only one, and then the others have to be compensated by pseudocritical velocities that may or may not be dominant, which, in turn, affects their influence on the instability lines. Second, especially for low damping levels, there are more asymptotes than these velocities predict. Third, there are cases where the instability lines intersect the lines of critical velocities of the moving force, which contradicts the regular behavior of the Winkler–Pasternak beam.

This paper is organized as follows: First, the model, along with its basic assumptions and simplifications, is presented in Section 2, where the governing equations are also given. The solutions obtained by the two analyzed methods, that is, the semi-analytical approach and the Green’s function method, are derived in Section 3 and Section 4, respectively. The set of dimensionless parameters necessary to describe the model are identified in Section 3. They are also used in Section 4, where some additional parameters are also introduced. Section 5 summarizes the range of allowable real railway track model parameters alongside the range of admissible dimensionless parameters. Parameters identifying a specific track section from the literature are also given. Several cases are selected to demonstrate the results. They refer to the specific track section as well as to other track sections identified by dimensionless parameters within the allowable intervals, including the damping of materials in the foundation. Finally, the two solution methods are compared. The main conclusions are drawn in Section 6. 

## 2. Mechanical Model and Governing Equations

As already mentioned in the Introduction, the three-viscoelastic-layer railway track model is widely used for its simplicity, numerical efficiency, and ability to effectively estimate the real behavior, as demonstrated in [71,72]. Its description was already given in previous works on layered models [51,52], but it is repeated here for completeness. In this model, the rail is modeled by a beam that is supported by linear spring–damper elements standing for rail pads, which are connected to point masses representing sleepers’ masses, which are, in turn, supported by linear spring–damper elements modeling the ballast, including its shear resistance. Further point masses represent the dynamically activated ballast mass, and these are supported by other linear spring–damper components modeling the foundation. This model is adapted for the analysis of transversal vibrations; therefore, the rail pad and foundation springs act only in the vertical direction, and the rotational degree of freedom is neglected. The shear resistance of the model is therefore introduced exclusively by the ballast shear stiffness elements. 

It would be more correct to introduce discrete supports according to the sleeper spacing; nevertheless, as indicated in the Introduction, discrete supports are only important at high frequencies, more precisely around the pinned–pinned value. Therefore, continuous supports are introduced in this paper because they are more suitable for semi-analytical derivations, as well as for derivations exploiting the dynamic Green’s function. Discrete supports are thus replaced by continuous ones. Such a simplification still provides a model that serves as a very good approximation of reality. It is also worthwhile to remark that small deflection variations between individual supports can equally be modeled by a harmonic component added to the constant moving force. 

**Figure 2:**
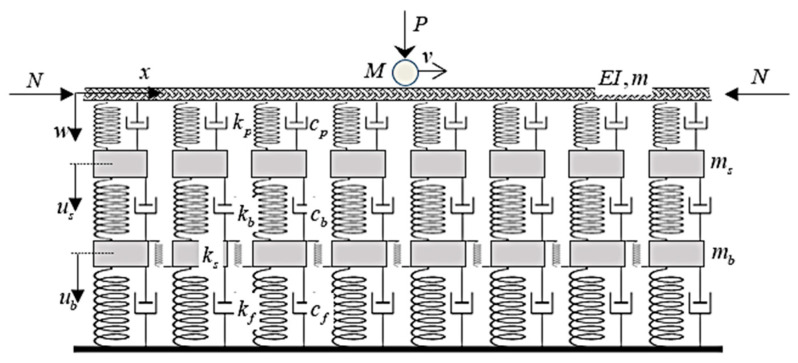
Railway track model with three viscoelastic layers subjected to an axial force and a uniformly moving mass subjected to a vertical force.

A constant mass is assumed to move over the model at a constant velocity, as depicted in Figure 2. The aim of the analysis is to determine the lowest velocity that marks the separation between stable and unstable behavior—the so-called critical velocity. The assumptions for such an analysis are listed below [51]:

(i) The beam is straight and prismatic, and it is made of isotropic homogeneous material.
(ii) The beam can withstand an axial force, in accordance with Figure 2.
(iii) The beam obeys the linear elastic Euler–Bernoulli theory.
(iv) Vertical displacements are measured from the equilibrium position corresponding to the deflection induced by the weight of the model components.
(v) The initial conditions are homogeneous; nevertheless, this has no effect on the critical velocity.
(vi) The velocity of the moving mass determines its horizontal position.
(vii) No friction acts at the contact point.
(viii) Loads and vertical displacements are assumed to be positive when acting downward.
(ix) As is usual in several applications, the acting force may or may not represent the moving mass weight.

In the semi-analytical approach, fixed (tight) contact between the mass and the beam is assumed, which means that the mass displacement and the displacement of the respective beam axis are always the same. An extension to the situation with a linear contact spring can be easily implemented in conformity with previous works [38], but it is not presented in this paper for simplicity. The Green’s function method, on the other hand, has better numerical stability when a contact spring is implemented, as will be demonstrated in Section 5.

The equations of motion can be written as follows [52]:

(1) $$EIw_{,xxxx}+mw_{,tt}+Nw_{,xx}+c_{p}\left(w_{,t}-u_{s,t}\right)+k_{p}\left(w-u_{s}\right)=\;p\left(x,t\right),$$

(2) $$m_{s}u_{s,tt}-c_{p}\left(w_{,t}-u_{s,t}\right)-k_{p}\left(w-u_{s}\right)+c_{b}\left(u_{s,t}-u_{b,t}\right)+k_{b}\left(u_{s}-u_{b}\right)=0,$$

(3) $$m_{b}u_{b,tt}-k_{s}u_{b,xx}-c_{b}\left(u_{s,t}-u_{b,t}\right)-k_{b}\left(u_{s}-u_{b}\right)+c_{f}u_{b,t}+k_{f}u_{b}=0,$$

where *p*(*x*, *t*) is the load, and *w*(*x*, *t*), *u_s_*(*x*, *t*), and *u_b_*(*x*, *t*) are the unknown deflections of the beam, the sleepers’ masses, and the ballast’s masses, respectively. *EI* and *m* stand for the bending stiffness and mass per unit length of the beam, and *N* is the axial force. *k_p_*, *c_p_*, *k_b_*, *c_b_*, and *k_f_*, *c_f_* are stiffness and viscous damping parameters related to the rail pads, ballast, and foundation, respectively. *k_s_* models the ballast shear stiffness, and *m_s_* and *m_b_* are the sleepers’ and ballast’s point masses. *x* is the fixed spatial coordinate, and *t* is the time. Derivatives are designated by the variable in the subscript position, preceded by a comma. 

In this formulation, all parameters are assumed to be distributed. To achieve this, the discrete values are divided by the sleepers’ spacing *l_s_*, except for the shear stiffness, which is modeled by a vertical spring, and therefore, a uniform distribution has to be applied to the inverse value. Therefore, if a discrete value of ${\bar{k}}_{s}$ with the unit N/m is used, then the distributed value is $k_{s}={\bar{k}}_{s}l_{s}$ with the unit N. 

As already mentioned, a constant mass *M* and a constant force *P* move with the same uniform velocity *v*. Then, the load *p*(*x*, *t*) is given by

(4) $$p\left(x,t\right)=\left(P-Mw_{0,tt}\left(t\right)\right)\delta \left(x-vt\right),$$

where δ is the Dirac delta function, and *w*_0_(*t*) is the displacement of the mass. 

The initial conditions are considered homogeneous, as already specified in assumption (v), and the boundary conditions state that the deflection and its slope vanish for x→±∞. 

Given the assumption of fixed contact, which states that $w_{0}\left(t\right)=w\left(x=vt,t\right)$, the chain rule can be used to remove *w*_0,*tt*_(*t*) from Equation (4):

(5) $$p\left(x,t\right)=\left[P-M\left(w_{,tt}\left(x,t\right)+2vw_{,xt}\left(x,t\right)+v^{2}w_{,xx}\left(x,t\right)\right)\right]\delta \left(x-vt\right).$$

## 3. Semi-Analytical Approach

### 3.1. Solution of the Governing Equations

In order to obtain the deflection fields, firstly, it is convenient to switch the fixed spatial coordinate to a moving one, defined by *r = x − vt*; then [52],

(6) $$\begin{array}{l} EIw_{,rrrr}+m\left(w_{,tt}-2vw_{,rt}+v^{2}w_{,rr}\right)+Nw_{,rr}\\ +c_{p}\left(w_{,t}-vw_{,r}-u_{s,t}+vu_{s,r}\right)+k_{p}\left(w-u_{s}\right)=\;\;\left(P-Mw_{,tt}\right)\delta \left(r\right), \end{array}$$

(7) $$\begin{array}{l} m_{s}\left(u_{s,tt}-2vu_{s,rt}+v^{2}u_{s,rr}\right)-c_{p}\left(w_{,t}-vw_{,r}-u_{s,t}+vu_{s,r}\right)\\ -k_{p}\left(w-u_{s}\right)+c_{b}\left(u_{s,t}-vu_{s,r}-u_{b,t}+vu_{b,r}\right)+k_{b}\left(u_{s}-u_{b}\right)=0, \end{array}$$

(8) $$\begin{array}{l} m_{b}\left(u_{b,tt}-2vu_{b,rt}+v^{2}u_{b,rr}\right)-k_{s}u_{b,rr}-c_{b}\left(u_{s,t}-vu_{s,r}-u_{b,t}+vu_{b,r}\right)\\ -k_{b}\left(u_{s}-u_{b}\right)+c_{f}\left(u_{b,t}-vu_{b,r}\right)+k_{f}u_{b}=0. \end{array}$$

Further, dimensionless parameters are introduced because all results will be presented in a dimensionless form. Actual track-specific data are also used, but, in general, all parameters are considered within the range of allowable values that will be defined in Section 5.

A Winkler beam with bending stiffness *EI*, mass per unit length *m*, and foundation stiffness *k_f_* is chosen as a reference. Then:

(9) $$\chi =\sqrt[{4}]{\frac{k_{f}}{4EI}},v_{ref}=\sqrt[{4}]{\frac{4k_{f}EI}{m^{2}}}=\frac{1}{\chi }\sqrt{\frac{k_{f}}{m}},\;\alpha =\frac{v}{v_{ref}},$$

where *v_ref_* is the critical velocity of the force moving over the reference beam, and α stands for the velocity ratio. The dimensionless spatial coordinate and time follow as

(10) $$\xi =\chi r,\;\tau =\chi v_{ref}t.$$

Displacement fields are related to the maximum static deflection induced by force *P* on the reference beam; thus,

(11) $$w_{st}=\frac{P\chi }{2k_{f}},\;\tilde{w}=\frac{w}{w_{st}},\;{\tilde{u}}_{s}=\frac{u_{s}}{w_{st}},\;{\tilde{u}}_{b}=\frac{u_{b}}{w_{st}},$$

The mass and stiffness ratios are

(12) $$\mu_{s}=\frac{m_{s}}{m},\mu_{b}=\frac{m_{b}}{m},\;\kappa_{p}=\frac{k_{p}}{k_{f}},\;\kappa_{b}=\frac{k_{b}}{k_{f}},$$

the damping ratios read

(13) $$\eta_{p}=\frac{c_{p}}{2\sqrt{mk_{f}}},\eta_{b}=\frac{c_{b}}{2\sqrt{mk_{f}}},\;\eta_{f}=\frac{c_{f}}{2\sqrt{mk_{f}}},$$

and the axial force and shear ratios are

(14) $$\eta_{N}=\frac{N}{2\sqrt{k_{f}EI}},\;\eta_{s}=\frac{k_{s}}{2\sqrt{k_{f}EI}}.$$

Finally, the moving mass and force ratios are

(15) $$\eta_{M}=\frac{M\chi }{m},\;\eta_{C}=\frac{P}{P}.$$

Defining the moving force is certainly abundant, but it is mathematically more correct and lends itself to further generalizations.

Using the definitions above, one obtains the following:

(16) $$\begin{array}{l} {\tilde{w}}_{,\xi \xi \xi \xi }+4\left({\tilde{w}}_{,\tau \tau }-2\alpha {\tilde{w}}_{,\xi \tau }+\alpha^{2}{\tilde{w}}_{,\xi \xi }\right)+4\eta_{N}{\tilde{w}}_{,\xi \xi }+8\eta_{p}\left({\tilde{w}}_{,\tau }-\alpha {\tilde{w}}_{,\xi }-{\tilde{u}}_{s,\tau }+\alpha {\tilde{u}}_{s,\xi }\right)\\ +4\kappa_{p}\left(\tilde{w}-{\tilde{u}}_{s}\right)=\left(8\eta_{C}-4\eta_{M}{\tilde{w}}_{,\tau \tau }\right)\delta \left(\xi \right) \end{array}$$

(17) $$\begin{array}{l} \mu_{s}\left({\tilde{u}}_{s,\tau \tau }-2\alpha {\tilde{u}}_{s,\xi \tau }+\alpha^{2}{\tilde{u}}_{s,\xi \xi }\right)-2\eta_{p}\left({\tilde{w}}_{,\tau }-\alpha {\tilde{w}}_{,\xi }-{\tilde{u}}_{s,\tau }+\alpha {\tilde{u}}_{s,\xi }\right)\\ -\kappa_{p}\left(\tilde{w}-{\tilde{u}}_{s}\right)+2\eta_{b}\left({\tilde{u}}_{s,\tau }-\alpha {\tilde{u}}_{s,\xi }-{\tilde{u}}_{b,\tau }+\alpha {\tilde{u}}_{b,\xi }\right)+\kappa_{b}\left({\tilde{u}}_{s}-{\tilde{u}}_{b}\right)=0 \end{array}$$

(18) $$\begin{array}{l} \mu_{b}\left({\tilde{u}}_{b,\tau \tau }-2\alpha {\tilde{u}}_{b,\xi \tau }+\alpha^{2}{\tilde{u}}_{b,\xi \xi }\right)-\eta_{s}{\tilde{u}}_{b,\xi \xi }-2\eta_{b}\left({\tilde{u}}_{s,\tau }-\alpha {\tilde{u}}_{s,\xi }-{\tilde{u}}_{b,\tau }+\alpha {\tilde{u}}_{b,\xi }\right)\\ -\kappa_{b}\left({\tilde{u}}_{s}-{\tilde{u}}_{b}\right)+2\eta_{f}\left({\tilde{u}}_{b,\tau }-\alpha {\tilde{u}}_{b,\xi }\right)+{\tilde{u}}_{b}=0 \end{array}$$

For the stability analysis, the Laplace transform must be applied first to capture the initial instant because the transient part of the vibrations is essential:

(19) $$\tilde{W}\left(\xi ,\bar{q}\right)=\int_{0}^{\infty }\tilde{w}\left(\xi ,\tau \right)e^{-\bar{q}\tau }d\tau ,\tilde{V}\left(\xi ,\bar{q}\right)=\int_{0}^{\infty }{\tilde{u}}_{s}\left(\xi ,\tau \right)e^{-\bar{q}\tau }d\tau ,\;\;\tilde{U}\left(\xi ,\bar{q}\right)=\int_{0}^{\infty }{\tilde{u}}_{b}\left(\xi ,\tau \right)e^{-\bar{q}\tau }d\tau .$$

In fact, $\bar{q}=iq$, but Equation (19) is used to keep the formalism of the Laplace transform. For homogeneous initial conditions, one obtains

(20) $$\begin{array}{l} {\tilde{W}}_{,\xi \xi \xi \xi }+4\left(-q^{2}\tilde{W}-2iq\alpha {\tilde{W}}_{,\xi }+\alpha^{2}{\tilde{W}}_{,\xi \xi }\right)+4\eta_{N}{\tilde{W}}_{,\xi \xi }+8\eta_{p}\left(iq\tilde{W}-\alpha {\tilde{W}}_{,\xi }-iq\tilde{V}+\alpha {\tilde{V}}_{,\xi }\right)\\ +4\kappa_{p}\left(\tilde{W}-\tilde{V}\right)=\;\left(\frac{8\eta_{C}}{iq}+4\eta_{M}q^{2}\tilde{W}\right)\delta \left(\xi \right) \end{array}$$

(21) $$\begin{array}{l} \mu_{s}\left(-q^{2}\tilde{V}-2iq\alpha {\tilde{V}}_{,\xi }+\alpha^{2}{\tilde{V}}_{,\xi \xi }\right)-2\eta_{p}\left(iq\tilde{W}-\alpha {\tilde{W}}_{,\xi }-iq\tilde{V}+\alpha {\tilde{V}}_{,\xi }\right)\\ -\kappa_{p}\left(\tilde{W}-\tilde{V}\right)+2\eta_{b}\left(iq\tilde{V}-\alpha {\tilde{V}}_{,\xi }-iq\tilde{U}+\alpha {\tilde{U}}_{,\xi }\right)+\kappa_{b}\left(\tilde{V}-\tilde{U}\right)=0 \end{array}$$

(22) $$\begin{array}{l} \mu_{b}\left(-q^{2}\tilde{U}-2iq\alpha {\tilde{U}}_{,\xi }+\alpha^{2}{\tilde{U}}_{,\xi \xi }\right)-\eta_{s}{\tilde{U}}_{,\xi \xi }-2\eta_{b}\left(iq\tilde{V}-\alpha {\tilde{V}}_{,\xi }-iq\tilde{U}+\alpha {\tilde{U}}_{,\xi }\right)\\ -\kappa_{b}\left(\tilde{V}-\tilde{U}\right)+2\eta_{f}\left(iq\tilde{U}-\alpha {\tilde{U}}_{,\xi }\right)+\tilde{U}=0 \end{array}$$

Then, the Fourier transform is applied as follows:

(23) $$F\left(p,\bar{q}\right)=\int_{-\infty }^{\infty }\tilde{F}\left(\xi ,\bar{q}\right)e^{-ip\xi }d\xi .$$

Without losing generality, functional dependence can be adapted to *q* instead of $\bar{q}$, leading to

(24) $$\begin{array}{l} W\left(p,q\right)\left[p^{4}-4{\left(q-\alpha p\right)}^{2}-4\eta_{N}p^{2}+8{i\eta }_{p}\left(q-\alpha p\right)+4\kappa_{p}\right]\\ -V\left(p,q\right)\left[8{i\eta }_{p}\left(q-\alpha p\right)+4\kappa_{p}\right]=\frac{8\eta_{C}}{iq}+4\eta_{M}q^{2}\tilde{W}\left(0,q\right) \end{array}$$

(25) $$\begin{array}{l} -W\left(p,q\right)\left[2{i\eta }_{p}\left(q-\alpha p\right)+\kappa_{p}\right]\\ +V\left(p,q\right)\left[-\mu_{s}{\left(q-\alpha p\right)}^{2}+2{i\eta }_{p}\left(q-\alpha p\right)+\kappa_{p}+2{i\eta }_{b}\left(q-\alpha p\right)+\kappa_{b}\right]\\ -U\left(p,q\right)\left[2{i\eta }_{b}\left(q-\alpha p\right)+4\kappa_{b}\right]=0 \end{array}$$

(26) $$\begin{array}{l} -V\left(p,q\right)\left[2{i\eta }_{b}\left(q-\alpha p\right)+\kappa_{b}\right]\\ +U\left(p,q\right)\left[-\mu_{b}{\left(q-\alpha p\right)}^{2}+\eta_{s}p^{2}+2{i\eta }_{b}\left(q-\alpha p\right)+\kappa_{b}+2{i\eta }_{f}\left(q-\alpha p\right)+1\right]=0 \end{array}$$

The expressions introduced by Equations (24)–(26) can be simplified by introducing

(27) $$D_{1}\left(p,q\right)=p^{4}-4{\left(q-\alpha p\right)}^{2}-4\eta_{N}p^{2}$$

(28) $$D_{2}\left(p,q\right)=2{i\eta }_{p}\left(q-\alpha p\right)+\kappa_{p}$$

(29) $$D_{3}\left(p,q\right)=-\mu_{s}{\left(q-\alpha p\right)}^{2}$$

(30) $$D_{4}\left(p,q\right)=2{i\eta }_{b}\left(q-\alpha p\right)+\kappa_{b}$$

(31) $$D_{5}\left(p,q\right)=-\mu_{b}{\left(q-\alpha p\right)}^{2}+\eta_{s}p^{2}$$

(32) $$D_{6}\left(p,q\right)=2{i\eta }_{f}\left(q-\alpha p\right)+1$$

Then, Equations (24)–(26) can be written as (the dependence on the variables is omitted for simplicity, except for the term to be solved)

(33) $$\left[\begin{array}{ccc} D_{1}+4D_{2} & -4D_{2} & 0\\ -D_{2} & D_{3}+D_{2}+D_{4} & -D_{4}\\ 0 & -D_{4} & D_{5}+D_{4}+D_{6} \end{array}\right]\left\{\begin{array}{c} W\\ V\\ U \end{array}\right\}=\left\{\begin{array}{c} \frac{8\eta_{C}}{iq}+4\eta_{M}q^{2}\tilde{W}\left(0,q\right)\\ 0\\ 0 \end{array}\right\}.$$

In order to remove $\tilde{W}\left(0,q\right)$, firstly, Equation (33) is solved for *W*(*p*, *q*):

(34) $$W=\frac{d_{2}}{d_{3}}\left(\frac{8\eta_{C}}{iq}+4\eta_{M}q^{2}\tilde{W}\left(0,q\right)\right),$$

where *d*_3_ is the determinant of the system (33), and *d*_2_ = (*D*_2_ + *D*_3_)·(*D*_4_ + *D*_5_ + *D*_6_) + *D*_4_ · (*D*_5_ + *D*_6_) is the subdeterminant of order 2, obtained as a determinant of the matrix of Equation (33) with the first line and column cut.

Now, one can perform the inverse Fourier transform to recover the Laplace image:

(35) $$\tilde{F}\left(\xi ,q\right)=\frac{1}{2\pi }\int_{-\infty }^{\infty }F\left(p,q\right)e^{ip\xi }dp$$

(36) $$\tilde{W}\left(\xi ,q\right)=\frac{1}{2\pi }\left[\frac{8\eta_{C}}{iq}+4\eta_{M}q^{2}\tilde{W}\left(0,q\right)\right]\int_{-\infty }^{\infty }\frac{d_{2}}{d_{3}}e^{ip\xi }dp.$$

Then, ξ=0 can be substituted, and by introducing

(37) $$K\left(\xi ,q\right)=\int_{-\infty }^{\infty }\frac{d_{2}}{d_{3}}e^{ip\xi }dp,$$

one obtains

(38) $$\tilde{W}\left(0,q\right)=\frac{4\eta_{C}}{iq\left(\pi -2\eta_{M}q^{2}K\left(0,q\right)\right)}K\left(0,q\right).$$

Substituting Equation (38) into Equation (36), one finally obtains

(39) $$\tilde{W}\left(\xi ,q\right)=\frac{4\eta_{C}}{iq\left(\pi -2\eta_{M}q^{2}K\left(0,q\right)\right)}K\left(\xi ,q\right).$$

It is worth noting that these derivations are conceptually the same as in previous works [10,52]; the only difference compared to other layered models is the definition of the function *K*(ξ, *q*), which is proportional to the equivalent flexibility of the supporting structure.

The final step lies in the inverse Laplace transform, which is defined by

(40) $$f\left(t\right)=\frac{1}{2\pi i}\underset{T-\infty }{\lim}\int_{a-iT}^{a+iT}e^{st}F\left(s\right)ds.$$

Likewise, as in previous works, it is found that it is more convenient to switch the real and imaginary axes; thus, the final deflection shape of the beam can be numerically evaluated as follows:

(41) $$\tilde{w}\left(\xi ,\tau \right)=\frac{1}{\pi }\int_{-ia-\infty }^{-ia+\infty }\frac{-{2i\eta }_{C}K\left(\xi ,q\right)}{q\left(\pi -2\eta_{M}q^{2}K\left(0,q\right)\right)}e^{iq\tau }dq,$$

where *a* > 0 must be chosen so that all discontinuities and poles of the function to be integrated lie above the line (−i*a* − ∞, i*a* + ∞) in the complex *q*-plane. 

With the help of contour integration methods, the following can be derived:

(42) $$\tilde{w}\left(\xi ,\tau \right)=\sum \mathrm{res}\left(q,i\tilde{W}\left(\xi ,q\right)e^{iq\tau }\right)+{\tilde{w}}_{tr}\left(\xi ,\tau \right),$$

where ${\overset{⌢}{w}}_{tr}\left(\xi ,\tau \right)$ is the truly transient part, and res designates the residue. Then,

(43) $$\tilde{w}\left(\xi ,\tau \right)=\frac{4\eta_{C}K\left(\xi ,0\right)}{\pi }-\sum_{j}\frac{2\eta_{C}K\left(\xi ,q_{M_{j}}\right)}{\pi +\eta_{M}q_{M_{j}}^{3}K_{,q}\left(0,q_{M_{j}}\right)}e^{iq_{M_{j}}\tau }+{\tilde{w}}_{tr}\left(\xi ,\tau \right),$$

where the first term stands for the steady-state solution, and $q_{M_{j}}$ in the second term represents mass-induced frequencies as solutions of the characteristic equation. Methods for the determination of mass-induced frequencies and their properties are detailed in previous works [10,38,59]. K(ξ,0), $K\left(\xi ,q_{M_{j}}\right)$, and $K_{,q}\left(0,q_{M_{j}}\right)$ can be evaluated accurately by contour integration and analytical terms for residues of simple and double poles.

Therefore, contour integration is used in two stages. One instance is for *K* and *K_,q_* calculations in the *p*-complex plane for a given *q*. In this case, the *p*-poles can be determined numerically, and the residues can be obtained analytically. The other usage is more complicated, because it relates to evaluation in the *q*-complex plane to derive Equation (43). To achieve this, the *q*-poles of the function to be integrated in Equation (41) must be determined. It is clear from Equation (41) that one pole is obvious, that is, 0, and defines the steady-state part of the solution. The other poles, $q_{M_{j}}$, are solutions of the characteristic equation π − 2η*_M_q*^2^*K*(0, *q*) = 0. They are referred to as mass-induced frequencies, and the associated residues identify the harmonic part of the transient vibration. The last part of Equation (43), ${\tilde{w}}_{tr}\left(\xi ,\tau \right)$, also belongs to the transient part of the solution and is obtained numerically. The reason for its existence is that the *K*-function has step discontinuities of bounded values. For contour integration, branch cuts have to be introduced to remove such locations from the complex *q*-plane. Integration along branch cuts leads to ${\tilde{w}}_{tr}\left(\xi ,\tau \right)$. While ${\tilde{w}}_{tr}\left(\xi ,\tau \right)$ is generally less important for one- or two-layer models, unfortunately, this is not the case for three-layer models. There are many regions where $q_{M_{j}}$ does not exist, and then the sum of residues is insufficient to approximate the full solution. Therefore, a numerical calculation according to Equation (41) must usually be performed.

### 3.2. Critical Velocity of a Moving Mass

The critical velocity of the moving mass is defined here as the lowest α that marks the separation between stable and unstable regions. An important finding in the semi-analytical approach is that the discontinuity lines as a function of *q* are always located at positions with the *q* of positive imaginary parts, and Equation (43) implies that instability occurs when the imaginary part of $q_{M_{j}}$ is negative. Hence, for the stability analysis, which is the goal of this paper, it is not important to analyze ${\tilde{w}}_{tr}\left(\xi ,\tau \right)$ or to determine the discontinuity lines. Instability lines are identified by real-valued poles $q_{M_{j}}$, since, in the damped case, assuming all problem parameters are fixed and taking $q_{M_{j}}$ as a function of α, this necessarily implies switching between $q_{M_{j}}$ with positive and negative imaginary parts. These values are not affected by the discontinuity lines, and thus, their tracing as a function of α is not interrupted. They can be determined in a very simple way. Taking the characteristic equation π − 2η*_M_q*^2^*K*(0, *q*) = 0, it is clear that it suffices to test *K*(0, *q*) as a function of α and find a real *q* for which *K*(0, *q*) is also real. This can be realized by simple iteration on the real *q*-axis. All such positions can be determined without any doubt that some value was missed. Calculations can be performed with very high accuracy using the exact value of *K*(0, *q*) obtained semi-analytically by contour integration. The corresponding η*_M_* is then calculated. Further, all branches are plotted on the α-η*_M_* plane.

Instability lines therefore mark the separation between $q_{M_{j}}$ with positive and negative imaginary parts, meaning between stable and unstable behavior, or between two unstable regions with different degrees of instability. These cases can be easily distinguished from the graphs of branches of instability lines. Then, for any selected η*_M_*, it is possible to identify α-intervals where the system is unstable. This is a simple task compared to searching for $q_{M_{j}}$ in general, which implies solving the complex roots of a complex equation. 

### 3.3. Critical Velocity of a Moving Force

In order to analyze the results indicated in the previous section, it is important to know the critical velocities of the structure, i.e., the critical velocities that can be assigned to one moving constant force, because there is a tight relationship between the critical velocities of a moving force and a moving mass. 

The critical velocity of the moving force can be determined by analyzing the steady-state deflection. When the force is passing over the structure at a critical velocity, then resonance occurs in the undamped case, and the deflection tends to infinity. 

For the steady-state analysis, the time dependence can be removed. In addition, the loading term is reduced to one moving constant force, and thus, the previous Equations (16)–(18) simplify to

(44) $${\tilde{w}}_{,\xi \xi \xi \xi }+4\eta_{N}{\tilde{w}}_{,\xi \xi }+4\alpha^{2}{\tilde{w}}_{,\xi \xi }-8\alpha \eta_{p}\left({\tilde{w}}_{,\xi }-{\tilde{u}}_{s,\xi }\right)+4\kappa_{p}\left(\tilde{w}-{\tilde{u}}_{s}\right)=8\eta_{C}\delta \left(\xi \right),$$

(45) $$\alpha^{2}\mu_{s}{\tilde{u}}_{s,\xi \xi }+2\alpha \eta_{p}\left({\tilde{w}}_{,\xi }-{\tilde{u}}_{s,\xi }\right)-\kappa_{p}\left(\tilde{w}-{\tilde{u}}_{s}\right)-2\alpha \eta_{b}\left({\tilde{u}}_{s,\xi }-{\tilde{u}}_{b,\xi }\right)+\kappa_{b}\left({\tilde{u}}_{s}-{\tilde{u}}_{b}\right)=0,$$

(46) $$\alpha^{2}\mu_{b}{\tilde{u}}_{b,\xi \xi }+2\alpha \eta_{b}\left({\tilde{u}}_{s,\xi }-{\tilde{u}}_{b,\xi }\right)-\kappa_{b}\left({\tilde{u}}_{s}-{\tilde{u}}_{b}\right)-2\alpha \eta_{f}{\tilde{u}}_{b,\xi }+{\tilde{u}}_{b}-\eta_{s}{\tilde{u}}_{b,\xi \xi }=0.$$

Then, in the Fourier space, by using Equation (23), one obtains the following:

(47) $$p^{4}W-4\eta_{N}p^{2}W-4\alpha^{2}p^{2}W-8ip\alpha \eta_{p}\left(W-V\right)+4\kappa_{p}\left(W-V\right)=8\eta_{C},$$

(48) $$-p^{2}\mu_{s}\alpha^{2}V+2ip\alpha \eta_{p}\left(W-V\right)-\kappa_{p}\left(W-V\right)-2ip\alpha \eta_{b}\left(V-U\right)+\kappa_{b}\left(V-U\right)=0,$$

(49) $$-p^{2}\mu_{b}\alpha^{2}U+2ip\alpha \eta_{b}\left(V-U\right)-\kappa_{b}\left(V-U\right)-2ip\alpha \eta_{f}U+U+\eta_{s}p^{2}U=0.$$

With the introduction of

(50) $$D_{1}^{st}\left(p\right)=\;p^{4}-4p^{2}\alpha^{2}-4p^{2}\eta_{N}$$

(51) $$D_{2}^{st}\left(p\right)=\;\kappa_{p}-2ip\alpha \eta_{p}$$

(52) $$D_{3}^{st}\left(p\right)=-p^{2}\mu_{s}\alpha^{2}$$

(53) $$D_{4}^{st}\left(p\right)=\kappa_{b}-2ip\alpha \eta_{b}$$

(54) $$D_{5}^{st}\left(p\right)=-p^{2}\mu_{b}\alpha^{2}+\eta_{s}p^{2}$$

(55) $$D_{6}^{st}\left(p\right)=1-2ip\alpha \eta_{f},$$

which is formally the same as in Equation (33), only all terms *q* − α*p* are simplified to −α*p*, and the loading term includes only the moving force:

(56) $$\left[\begin{array}{ccc} D_{1}^{st}+4D_{2}^{st} & -4D_{2}^{st} & 0\\ -D_{2}^{st} & D_{3}^{st}+D_{2}^{st}+D_{4}^{st} & -D_{4}^{st}\\ 0 & -D_{4}^{st} & D_{5}^{st}+D_{4}^{st}+D_{6}^{st} \end{array}\right]\left\{\begin{array}{c} W\\ V\\ U \end{array}\right\}=\left\{\begin{array}{c} 8\eta_{C}\\ 0\\ 0 \end{array}\right\}.$$

Thus,

(57) $$W=8\eta_{C}\frac{d_{2}^{st}}{d_{3}^{st}},$$

where $d_{3}^{st}$ and $d_{2}^{st}$ have the same meaning as for the moving mass, only the terms that are involved are adapted to the steady-state situation, as indicated by the superscript. Contour integration can be used to accurately calculate deflection shapes, avoiding the numerical problems inherent to the FFT, including the necessity of introducing damping for numerical stability. 

To identify the critical velocity, it is necessary to find a double real *p*-root of $d_{3}^{st}$ in the undamped case and the corresponding $\alpha_{cr}^{st}$, that is, to find the real pair $\left(p,\alpha_{cr}^{st}>0\right)$ that solves $d_{3}^{st}=0$ and $d_{3,p}^{st}=0$ when η*_p_* = η*_b_* = η*_f_* = 0. It is easy to verify that for η*_p_* = η*_b_* = η*_f_* = 0, $d_{3}^{st}$ is a cubic polynomial for $\bar{\alpha }=\alpha^{2}$ and a fourth-order polynomial for $\bar{p}=p^{2}$ with real coefficients. α is real and positive, and so is $\overset{-}{\alpha }$. *p* is real and $\bar{p}$ is therefore real and positive because zero values are excluded. $d_{3,p}^{st}$ can be divided by 2*p*, and then the resulting expression is still a cubic polynomial for $\bar{\alpha }=\alpha^{2}$ and a cubic polynomial for $\bar{p}=p^{2}$. Simplifications like in [35] are not possible; however, it is possible to numerically solve the exact value of $\alpha_{cr}^{st}$. It is also possible to show that there can only be 1, 3, or 5 valid pairs $\left(p,\alpha_{cr}^{st}\right)$. To find the roots in the simpler case where η*_N_* = η*_s_* = 0, it is possible to consider $\bar{p}d_{3,p}^{st}/(2p)-3d_{3}^{st}$, which is a quadratic polynomial for $\overset{-}{\alpha }$. Its two solutions can be introduced into $d_{3}^{st}$ or $d_{3,p}^{st}/(2p)$. In both cases, all $\bar{p}$-roots can be obtained directly with Maple’s “solve” function. If necessary, the number of digits can be increased, but no additional numerical methods are needed. Real roots are always paired with their opposite values, and complex roots exist with all four combinations of signs. Only positive real $\bar{p}$ stays for a valid solution, and only this value yields a positive $\overset{-}{\alpha }$. Since complex roots are always in groups of four, there can be 2, 6, or 10 real roots, except in cases with multiple roots. It is then found that there are 1, 3, or 5 resonant velocities. 

### 3.4. Long Finite Beam—Eigenmode Expansion

It is also advantageous to perform the analysis on long finite beams because all results presented for infinite beams can be validated on long finite beams, as demonstrated in previous works [10,34,35,52]. It was proven that simple supports can be introduced, but the load should start to act a little farther from the support. In the context of this paper, it is important to analyze the critical velocities of the moving force, because this analysis allows identifying the so-called false critical velocity. 

To carry out such an analysis, the vibration modes and orthogonality conditions must be determined. In order to keep the analysis in the real domain, undamped modes are considered. Free undamped vibrations in fixed coordinates fulfill the following:

(58) $${\tilde{w}}_{,\xi \xi \xi \xi }+4\eta_{N}{\tilde{w}}_{,\xi \xi }+4{\tilde{w}}_{,\tau \tau }+4\kappa_{p}\left(\tilde{w}-{\tilde{u}}_{s}\right)=0$$

(59) $$\mu_{s}{\tilde{u}}_{s,\tau \tau }-\kappa_{p}\left(\tilde{w}-{\tilde{u}}_{s}\right)+\kappa_{b}\left({\tilde{u}}_{s}-{\tilde{u}}_{b}\right)=0$$

(60) $$\mu_{b}{\tilde{u}}_{b,\tau \tau }-\kappa_{b}\left({\tilde{u}}_{s}-{\tilde{u}}_{b}\right)+{\tilde{u}}_{b}-\eta_{s}{\tilde{u}}_{b,\xi \xi }=0$$

Admitting harmonic vibrations $\tilde{w}\left(\xi ,\tau \right)=\tilde{w}\left(\xi \right)e^{i\varpi \tau }$, ${\tilde{u}}_{s}\left(\xi ,\tau \right)={\tilde{u}}_{s}\left(\xi \right)e^{i\varpi \tau }$, and ${\tilde{u}}_{b}\left(\xi ,\tau \right)={\tilde{u}}_{b}\left(\xi \right)e^{i\varpi \tau }$, one obtains the following:

(61) $${\tilde{w}}_{,\xi \xi \xi \xi }+4\eta_{N}{\tilde{w}}_{,\xi \xi }-4\varpi^{2}\tilde{w}+4\kappa_{p}\left(\tilde{w}-{\tilde{u}}_{s}\right)=0$$

(62) $$-\mu_{s}\varpi^{2}{\tilde{u}}_{s}-\kappa_{p}\left(\tilde{w}-{\tilde{u}}_{s}\right)+\kappa_{b}\left({\tilde{u}}_{s}-{\tilde{u}}_{b}\right)=0$$

(63) $$-\mu_{b}\varpi^{2}{\tilde{u}}_{b}-\kappa_{b}\left({\tilde{u}}_{s}-{\tilde{u}}_{b}\right)+{\tilde{u}}_{b}-\eta_{s}{\tilde{u}}_{b,\xi \xi }=0$$

Then, assuming $\tilde{w}\left(\xi \right)=We^{ip\xi }$, ${\tilde{u}}_{s}\left(\xi \right)=Ve^{ip\xi }$, and ${\tilde{u}}_{b}\left(\xi \right)=Ue^{ip\xi }$ yields the following:

(64) $$p^{4}W-4p^{2}\eta_{N}W-4\varpi^{2}W+4\kappa_{p}\left(W-V\right)=0$$

(65) $$-\mu_{s}\varpi^{2}V-\kappa_{p}\left(W-V\right)+\kappa_{b}\left(V-U\right)=0$$

(66) $$-\mu_{b}\varpi^{2}U-\kappa_{b}\left(V-U\right)+U+\eta_{s}p^{2}U=0$$

which can be written in a matrix form:

(67) $$\left(\left[\begin{array}{ccc} \;p^{4}-4p^{2}\eta_{N}+4\kappa_{p} & -4\kappa_{p} & 0\\ -\kappa_{p} & \kappa_{p}+\kappa_{b} & -\kappa_{b}\\ 0 & -\kappa_{b} & \kappa_{b}+1+\eta_{s}p^{2} \end{array}\right]-\varpi^{2}\left[\begin{array}{ccc} \;4 & 0 & 0\\ 0 & \mu_{s} & 0\\ 0 & 0 & \mu_{b} \end{array}\right]\right)\left\{\begin{array}{c} W\\ V\\ U \end{array}\right\}=\left\{\begin{array}{c} 0\\ 0\\ 0 \end{array}\right\}.$$

The nullity of the determinant can be used to solve for the natural frequencies. For the given data and admitting that *L* is the beam length, *p* = *jπ*/(*L*χ) can be used owing to the simple supports. For the analysis presented here, it is better to keep *p*. The determinant is a cubic equation for ϖ^2^:

(68) $$\varpi^{6}-\bar{b}\varpi^{4}+\bar{c}\varpi^{2}-\bar{d}=0,$$

where

(69) $$\bar{b}=\bar{f}+\frac{\bar{g}}{\mu_{b}}+\frac{\kappa_{p}+\kappa_{b}}{\mu_{s}},\;\;\;\;\;\;\;\;\;\;\bar{c}=\frac{\bar{f}\bar{g}}{\mu_{b}}+\bar{f}\frac{\kappa_{p}+\kappa_{b}}{\mu_{s}}+\bar{g}\frac{\kappa_{p}+\kappa_{b}}{\mu_{b}\mu_{s}}-\frac{\kappa_{p}^{2}}{\mu_{s}}-\frac{\kappa_{b}^{2}}{\mu_{b}\mu_{s}},\;\;\;\;\;\;\bar{d}=\bar{f}\bar{g}\frac{\kappa_{p}+\kappa_{b}}{\mu_{b}\mu_{s}}-\bar{f}\frac{\kappa_{b}^{2}}{\mu_{s}\mu_{b}}-\bar{g}\frac{\kappa_{p}^{2}}{\mu_{s}\mu_{b}},\;\bar{f}=\frac{p^{4}}{4}-\eta_{N}p^{2}+\kappa_{p},\;\bar{g}=\kappa_{b}+\eta_{s}p^{2}+1.$$

Therefore, Equation (68) has an analytical solution that gives three simple real values for ϖ^2^ when the discriminant is positive, which is always true due to the physical nature of such an equation. Owing to its length, the analytical solution is not included here.

The resonance condition expresses the equality of excitation and natural frequencies:

(70) pα=ϖ.

Then, the critical velocity should correspond to α at a local minimum. The stationary condition

(71) $$\frac{d}{dp}\frac{\varpi \left(p\right)}{p}=0$$

can be solved for *p*, but only real and positive solutions are valid solutions. It can be verified that each frequency indicates several stationary values, but at most, five of them identify valid *p* and α. Specifically, there are only one, three, or five valid pairs (α, *p*) at resonance. When ordered according to α, then the odd values identify local minima and thus the critical velocity, but the even values stand for local maxima, identifying the false critical velocity. The false critical velocity still induces a resonance in the sense that the steady-state solution tends to infinity, but the usual properties of the critical velocity are not preserved, and furthermore, these values have no influence on the instability lines. 

## 4. Green’s Function Method

In this section, the above problem is solved using the D-decomposition method by applying the Green’s function in the frequency domain. The verification of the obtained results is carried out by evaluating the time response of the system using a numerical method for calculating the convolution integral [70]. To avoid numerical instability caused by the fact that the mass/beam contact is perfectly rigid, a linear elastic element with sufficiently high stiffness is introduced so that it does not significantly affect the result regarding the critical velocity.

### 4.1. Stability Issue

Equations of motion (16)–(18) describe both the steady-state behavior caused by a static load and the perturbed one. Stability depends on a small free vibration around the equilibrium point, and therefore, the homogeneous equations of motion must be extracted from above in matrix form:

(72) $$L_{\xi ,\tau }\Delta \tilde{q}(\xi ,\tau )=\Delta {\tilde{q}}_{M}(\xi ,\tau ),$$

where **L_ξ_**_,τ_ stands for the matrix differential operator

(73) $$L_{\xi ,\tau }=\left[\begin{array}{ccc} L_{\xi ,\tau 11} & L_{\xi ,\tau 12} & 0\\ L_{\xi ,\tau 21} & L_{\xi ,\tau 22} & L_{\xi ,\tau 23}\\ 0 & L_{\xi ,\tau 32} & L_{\xi ,\tau 33} \end{array}\right]$$

with

(74) $$L_{\xi ,\tau ,11}=\partial_{\xi }^{4}+4(\eta_{N}+\alpha^{2})\partial_{\xi }^{2}+4\partial_{\tau }^{2}-8\alpha \partial_{\xi ,\tau }^{2}-8{\alpha \eta }_{p}\partial_{\xi }+8\eta_{p}\partial_{\tau }+4\kappa_{p},\;\;\;\;\;\;\;L_{\xi ,\tau ,12}=8{\alpha \eta }_{p}\partial_{\xi }-8\eta_{p}\partial_{\tau }-4\kappa_{p},\;\;\;\;\;\;\;L_{\xi ,\tau ,21}=2{\alpha \eta }_{p}\partial_{\xi }-2\eta_{p}\partial_{\tau }-\kappa_{p},\;\;\;\;L_{\xi ,\tau ,22}=\alpha^{2}\mu_{s}\partial_{\xi }^{2}+\mu_{s}\partial_{\tau }^{2}-2{\alpha \mu }_{s}\partial_{\xi ,\tau }^{2}-2{\alpha (\eta }_{p}+\eta_{b})\partial_{\xi }+2{(\eta }_{p}+\eta_{b})\partial_{\tau }+\kappa_{p}+\kappa_{b},\;\;\;\;\;\;\;L_{\xi ,\tau ,23}=2{\alpha \eta }_{b}\partial_{\xi }-2\eta_{b}\partial_{\tau }-\kappa_{b},\;L_{\xi ,\tau ,32}=L_{\xi ,\tau ,23},\;\;\;\;\;\;L_{\xi ,\tau ,33}=(\alpha^{2}\mu_{b}-\eta_{s})\partial_{\xi }^{2}+\mu_{b}\partial_{\tau }^{2}-2{\alpha \mu }_{b}\partial_{\xi ,\tau }^{2}-2{\alpha (\eta }_{b}+\eta_{f})\partial_{\xi }+2{(\eta }_{b}+\eta_{f})\partial_{\tau }+\kappa_{b}+1,$$

where

(75) $$\begin{array}{ccc} \partial_{\xi }^{n}=\frac{\partial^{n}}{\partial \xi^{n}}, & \partial_{\tau }^{n}=\frac{\partial^{n}}{\partial \tau^{n}} & \partial_{\xi \tau }^{2}=\frac{\partial^{2}}{\partial \xi \partial \tau } \end{array}$$

and

(76) $$\begin{array}{l} \Delta \tilde{q}(\xi ,\tau )={\left[\begin{array}{ccc} \Delta \tilde{w}(\xi ,\tau ) & \Delta {\tilde{u}}_{s}(\xi ,\tau ) & \Delta {\tilde{u}}_{b}(\xi ,\tau ) \end{array}\right]}^{t}\\ \Delta {\tilde{q}}_{M}(\xi ,\tau )={\left[\begin{array}{ccc} -4\eta_{M}\Delta {\tilde{w}}_{,\tau \tau }(0,\tau )\delta (\xi ) & 0 & 0 \end{array}\right]}^{t}. \end{array}$$

The quantities in Equation (76)

(77) $$\begin{array}{ccc} \Delta \tilde{w}(\xi ,\tau ), & \Delta {\tilde{u}}_{s}(\xi ,\tau ), & \Delta {\tilde{u}}_{b}(\xi ,\tau ) \end{array}$$

are the system perturbations around the steady-state position.

Next, the Laplace transform is applied for the dimensionless time coordinate:

(78) $$\Delta Q(\xi ,\bar{q})=\int_{0}^{\infty }\Delta \tilde{q}(\xi ,\tau )e^{-\bar{q}\tau }d\tau ,$$

where

(79) $$\Delta Q(\xi ,\bar{q})={\left[\begin{array}{ccc} \Delta W(\xi ,\bar{q}) & \Delta U_{s}(\xi ,\bar{q}) & \Delta U_{b}(\xi ,\bar{q}) \end{array}\right]}^{t},$$

and $\Delta W(\xi ,\bar{q})$, $\Delta U_{s}(\xi ,\bar{q})$, and $\Delta U_{b}(\xi ,\bar{q})$ are related to $\Delta \tilde{w}(\xi ,\tau )$, $\Delta {\tilde{u}}_{s}(\xi ,\tau )$, and $\Delta {\tilde{u}}_{b}(\xi ,\tau )$ via the Laplace transform. 

Equation (72) becomes

(80) $$L_{\xi ,\bar{q}}\Delta Q(\xi ,\bar{q})={\left[\begin{array}{ccc} -4\eta_{M}{\bar{q}}^{2}\Delta W(0,\bar{q})\delta (\xi ) & 0 & 0 \end{array}\right]}^{t},$$

where

(81) $$L_{\xi ,\bar{q}}=\left[\begin{array}{ccc} L_{\xi ,\bar{q}11} & L_{\xi ,\bar{q}12} & 0\\ L_{\xi ,\bar{q}21} & L_{\xi ,\bar{q}22} & L_{\xi ,\bar{q}23}\\ 0 & L_{\xi ,\bar{q}32} & L_{\xi ,\bar{q}33} \end{array}\right]$$

with

(82) $$\begin{array}{l} L_{\xi ,\bar{q},11}=D_{\xi }^{4}+4(\eta_{N}+\alpha^{2})D_{\xi }^{2}+4{\bar{q}}^{2}-8\alpha \bar{q}D_{\xi }-8{\alpha \eta }_{p}D_{\xi }+8\eta_{p}\bar{q}+4\kappa_{p},\\ L_{\xi ,\bar{q},12}=8{\alpha \eta }_{p}D_{\xi }-8\eta_{p}\bar{q}-4\kappa_{p},\\ L_{\xi ,\bar{q},21}=2{\alpha \eta }_{p}D_{\xi }-2\eta_{p}\bar{q}-\kappa_{p},\\ L_{\xi ,\bar{q},22}=\alpha^{2}\mu_{s}D_{\xi }^{2}+\mu_{s}{\bar{q}}^{2}-2{\alpha \mu }_{s}\bar{q}D_{\xi }-2{\alpha (\eta }_{p}+\eta_{b})D_{\xi }+2{(\eta }_{p}+\eta_{b})\bar{q}+\kappa_{p}+\kappa_{b},\\ L_{\xi ,\bar{q},23}=2{\alpha \eta }_{b}D_{\xi }-2\eta_{b}\bar{q}-\kappa_{b},\\ L_{\xi ,\bar{q},32}=L_{\xi ,\bar{q},23},\\ L_{\xi ,\bar{q},33}=(\alpha^{2}\mu_{b}-\eta_{s})D_{\xi }^{2}+\mu_{b}{\bar{q}}^{2}-2{\alpha \mu }_{b}\bar{q}D_{\xi }-2{\alpha (\eta }_{b}+\eta_{f})D_{\xi }+2{(\eta }_{b}+\eta_{f})\bar{q}+\kappa_{b}+1 \end{array}$$

and

(83) $$D_{\xi }^{n}=\frac{d^{n}}{{d\xi }^{n}}.$$

Multiplying Equation (80) by the adjoint operator $L_{\xi ,\bar{q}}^{*}$,

(84) $$L_{\xi ,\bar{q}}^{*}=\left[\begin{array}{ccc} L_{\xi ,\bar{q},22}L_{\xi ,\bar{q},33}-L_{\xi ,\bar{q},23}L_{\xi ,\bar{q},32} & -L_{\xi ,\bar{q},12}L_{\xi ,\bar{q},33} & L_{\xi ,\bar{q},12}L_{\xi ,\bar{q},23}\\ -L_{\xi ,\bar{q},21}L_{\xi ,\bar{q},33} & L_{\xi ,\bar{q},11}L_{\xi ,\bar{q},33} & -L_{\xi ,\bar{q},11}L_{\xi ,\bar{q},23}\\ L_{\xi ,\bar{q},21}L_{\xi ,\bar{q},32} & -L_{\xi ,\bar{q},11}L_{\xi ,\bar{q},32} & L_{\xi ,\bar{q},11}L_{\xi ,\bar{q},22}-L_{\xi ,\bar{q},12}L_{\xi ,\bar{q},21} \end{array}\right]$$

the result reads

(85) $$H_{\xi ,\bar{q}}E_{3}\Delta Q(\xi ,\bar{q})=L_{\xi ,\bar{q}}^{*}{\left[\begin{array}{ccc} -4\eta_{M}{\bar{q}}^{2}\Delta W(0,\bar{q})\delta (\xi ) & 0 & 0 \end{array}\right]}^{t}.$$

where $H_{\xi ,\bar{q}}$ is the differential operator acting upon the **ξ** coordinate,

(86) $$H_{\xi ,\bar{q}}=L_{\xi ,\bar{q},11}L_{\xi ,\bar{q},22}L_{\xi ,\bar{q},33}-L_{\xi ,\bar{q},11}L_{\xi ,\bar{q},23}L_{\xi ,\bar{q},32}-L_{\xi ,\bar{q},12}L_{\xi ,\bar{q},21}L_{\xi ,\bar{q},33}$$

or

(87) $$H_{\xi ,\bar{q}}=\sum_{j=0}^{8}h_{j}(\alpha ,\bar{q}{)D}_{\xi }^{8-j},$$

where $h_{j}(\alpha ,\bar{q})$ denotes coefficients depending on α and $\bar{q}$ coordinates, and E_3_ stands for the identity matrix of the third order. $h_{j}(\alpha ,\bar{q})$ coefficients are given in the Appendix A.

It can be observed that the first equation of the matrix form (85) is not dependent upon the other two:

(88) $$H_{\xi ,\bar{q}}\Delta W(\xi ,\bar{q})=-4\eta_{M}{\bar{q}}^{2}R_{\xi ,\bar{q}}\Delta W(0,\bar{q})\delta (\xi ),$$

where the differential operator $R_{\xi ,\bar{q}}$ has the following form:

(89) $$R_{\xi ,\bar{q}}=L_{\xi ,\bar{q},22}L_{\xi ,\bar{q},33}-L_{\xi ,\bar{q},23}L_{\xi ,\bar{q},32}$$

or

(90) $$R_{\xi ,\bar{q}}=\sum_{j=0}^{4}r_{j}(\alpha ,\bar{q}{)D}_{\xi }^{4-j},$$

where

(91) $$\begin{array}{l} r_{0}(\alpha ,\bar{q})=\alpha^{2}\mu_{s}(\alpha^{2}\mu_{b}-\eta_{s}),\\ r_{1}(\alpha ,\bar{q})=-2\alpha \left[\alpha^{2}\mu_{s}{(\mu }_{b}\bar{q}{+\eta }_{b}+\eta_{f})+(\alpha^{2}\mu_{b}-\eta_{s}){(\mu }_{s}\bar{q}+\eta_{p}+\eta_{b})\right],\\ r_{2}(\alpha ,\bar{q})=\alpha^{2}\mu_{s}\left(\mu_{b}{\bar{q}}^{2}+2{(\eta }_{b}+\eta_{f})\bar{q}+\kappa_{b}+1\right)+4\alpha^{2}{(\mu }_{s}\bar{q}+\eta_{p}+\eta_{b}){(\mu }_{b}\bar{q}{+\eta }_{b}+\eta_{f})+\\ (\alpha^{2}\mu_{b}-\eta_{s})\left(\mu_{s}{\bar{q}}^{2}+2{(\eta }_{p}+\eta_{b})\bar{q}+\kappa_{p}+\kappa_{b}\right)-4\alpha^{2}\eta_{b}^{2},\\ r_{3}(\alpha ,\bar{q})=-2\alpha \left[\begin{array}{l} {(\mu }_{s}s+\eta_{p}+\eta_{b})\left(\mu_{b}{\bar{q}}^{2}+2{(\eta }_{b}+\eta_{f})\bar{q}+\kappa_{b}+1\right)+\\ {(\mu }_{b}\bar{q}{+\eta }_{b}+\eta_{f})\left(\mu_{s}{\bar{q}}^{2}+2{(\eta }_{p}+\eta_{b})\bar{q}+\kappa_{p}+\kappa_{b}\right)-\\ 2\eta_{b}(2\eta_{b}\bar{q}-\kappa_{b}) \end{array}\right],\\ r_{4}(\alpha ,\bar{q})=\left(\mu_{s}{\bar{q}}^{2}+2{(\eta }_{p}+\eta_{b})\bar{q}+\kappa_{p}+\kappa_{b}\right)\left(\mu_{b}{\bar{q}}^{2}+2{(\eta }_{b}+\eta_{f})\bar{q}+\kappa_{b}+1\right)-{\left(2\eta_{b}\bar{q}-\kappa_{b}\right)}^{2}. \end{array}$$

The solution to Equation (88) can be obtained in terms of the Green’s function associated with the differential operator $H_{\xi ,\bar{q}}$:

(92) $$\Delta W(\xi ,\bar{q})=-4\eta_{M}{\bar{q}}^{2}\Delta W(0,\bar{q})\int_{-\infty }^{\infty }G_{H}(\xi ,\varepsilon )R_{\xi ,\bar{q}}\delta (\varepsilon )d\varepsilon .$$

Considering **ξ** = 0, one obtains

(93) $$\Delta W(0,\bar{q})\left[1+4\eta_{M}{\bar{q}}^{2}\int_{-\infty }^{\infty }G_{H}(0,\varepsilon )R_{\xi ,\bar{q}}\delta (\varepsilon )d\varepsilon \right]=0$$

or

(94) $$\Delta W(0,\bar{q})\left[1+4\eta_{M}{\bar{q}}^{2}\sum_{i=0}^{4}r_{i}(\alpha ,\bar{q}){\left(-1\right)}^{4-i}\frac{\partial^{4-i}G_{H}(0,0)}{\partial \varepsilon^{4-i}}\right]=0.$$

Generally speaking, $\Delta W(0,\bar{q})\neq 0$, and it is mandatory to have

(95) $$1+4\eta_{M}{\bar{q}}^{2}\sum_{i=0}^{4}r_{i}(\alpha ,\bar{q}){\left(-1\right)}^{4-i}\frac{\partial^{4-i}G_{H}(0,0)}{\partial \varepsilon^{4-i}}=0,$$

which represents the characteristic equation associated with the differential operator **L_ξ_**_,τ_.

The Green’s function *G*_H_(ξ,ε) is the solution of the following equation:

(96) $$H_{\xi ,\bar{q}}G_{H}(\xi ,\varepsilon )=\delta (\xi -\varepsilon ),$$

which can be constructed using the same method as in [50],

(97) $$G_{H}(\xi ,\varepsilon ,iq)=\{\begin{array}{cc} G_{H}^{-}(\xi ,\varepsilon ,iq)=\frac{1}{h_{0}}\sum_{i=1}^{n}\frac{e^{\lambda_{i}(\xi -\varepsilon )}}{\overset{8}{\underset{k=1\;k\neq i}{\Pi }}(\lambda_{k}-\lambda_{i})} & -\infty <\xi <\varepsilon \;\\ G_{H}^{+}(\xi ,\varepsilon ,iq)=-\frac{1}{h_{0}}\sum_{i=n+1}^{8}\frac{e^{\lambda_{i}(\xi -\varepsilon )}}{\overset{8}{\underset{k=1\;k\neq i}{\Pi }}(\lambda_{k}-\lambda_{i})} & \varepsilon <\xi <\infty , \end{array}$$

where λ*_i_* is the eigenvalue of the H_ξ,*s*_ operator, and Re λ*_i_* > 0 for *i* = 1, …, *n*, and Re λ*_i_* < 0 for *i* = *n* + 1, …, 8. All eigenvalues λ*_i_* are dependent on α because the coefficients of the H_ξ,*s*_ operator are dependent on α and also on $\bar{q}=iq$.

The characteristic Equation (95) can be written as

(98) $$Z(\alpha ,\bar{q})=1,$$

where $Z(\alpha ,\bar{q})$ is given by

(99) $$Z(\alpha ,\bar{q})=-4\frac{\eta_{M}{\bar{q}}^{2}}{h_{0}}\sum_{i=1}^{n}\frac{\sum_{j=0}^{4}r_{j}(\bar{q}{)\lambda }_{i}^{4-j}}{\overset{8}{\underset{k=1\;k\neq i}{\Pi }}(\lambda_{k}-\lambda_{i})}$$

**Figure 3:**
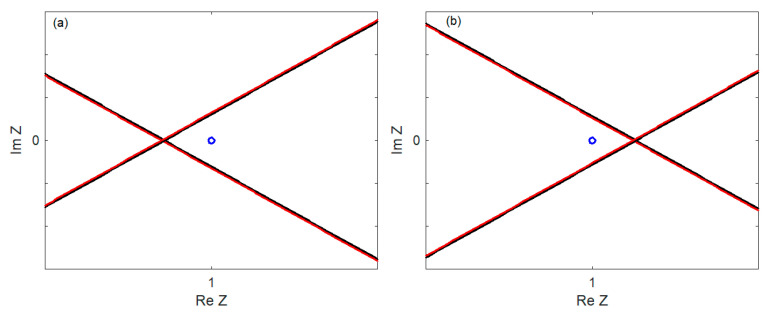
D-decomposition rule: (**a**) stable; (**b**) unstable; —, (Re *Z*(α, ±i*q*), Im *Z*(α, ±i*q*)); —, (Re *Z*(α, ζ ± i*q*), Im *Z*(α, ζ ± i*q*)); ⸰, point of coordinates (Re Z = 1, Im *Z* = 0).

**Figure 4:**
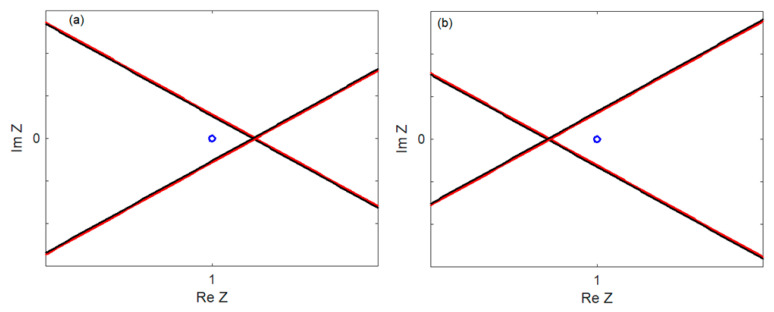
D-decomposition rule: (**a**) stable; (**b**) unstable;—, (Re *Z*(α, ±i*q*), Im *Z*(α, ±i*q*)); —, (Re *Z*(α, ζ ± i*q*), Im *Z*(α, ζ ± i*q*)); ⸰, point of coordinates (Re *Z* = 1, Im *Z* = 0).

The stability of the system can be determined based on the characteristic Equation (98) using the D-decomposition method. For a certain α velocity, the curve (Re *Z*(α, ±i*q*), Im *Z*(α, ±i*q*)) becomes the border between the stable and unstable parts of the complex plane, and the curve (Re *Z*(α, ζ ± i*q*), Im *Z*(α, ζ ± i*q*)) with ζ > 0 passes through an unstable part. When the point (1, 0) belongs to the stable part (see Figure 3a or Figure 4a), the system is stable. Figure 3b and Figure 4b show the position of the point (1, 0) when the system is unstable. Unstable part of the complex plane can be to the left of the curve (Re Z(α, ±i*q*), Im Z(α, ±i*q*)), as in Figure 3, or to the right, as in Figure 4.

### 4.2. Time-Domain Response

In this section, an algorithm for calculating the time series of a moving mass on an infinite beam on a three-layer viscoelastic foundation based on Green’s function theory and the convolution theorem is presented in terms of dimensionless quantities. 

To avoid numerical instability when the contact between the moving mass and the beam is rigid, an elastic element of stiffness *k_c_* is inserted between the moving mass and the beam. 

The nonhomogeneous term in Equation (1) becomes

(100) $$p(x,t)=P_{c}(t)\delta (x-Vt),$$

where *P_c_*(*t*) is the contact force between the moving mass and the beam. In addition, the equation of motion of the moving mass,

(101) $$M\ddot{z}(t)=P-P_{c}(t),$$

where *z*(*t*) is the vertical mass displacement, and the equation of the moving mass/beam contact

(102) $$P_{c}(t)=k_{c}\left(w(vt,t)-z(t)\right)H\left(w(vt,t)-z(t)\right),$$

where *H*(.) is the Heaviside step function, should be further considered. By introducing the Heaviside step function, the loss of contact between the moving mass and the beam is simulated.

Changing the reference frame, the nonhomogeneous term in Equation (6) becomes *P_c_*(*t*)δ(*r*), and the equation of the contact reads

(103) $$P_{c}(t)=k_{c}\left(w(0,t)-z(t)\right)H\left(w(0,t)-z(t)\right).$$

For rewriting in dimensionless quantities, three new dimensionless parameters must be introduced, in conformity with Equations (11), (12), and (15):

(104) $$\tilde{z}=\frac{z}{w_{st}},\;{\tilde{\eta }}_{P_{c}}=\frac{P_{c}}{P},\;\kappa_{c}=\frac{k_{c}\chi }{k_{f}}.$$

The interaction of a moving mass on an infinite beam on a three-layer viscoelastic foundation is then described by Equations (16)–(18), where the right-hand side of Equation (16) is adjusted to

(105) $$8{\tilde{\eta }}_{P_{c}}\delta \left(\xi \right)$$

and by Equations (101) and (103), which, in the dimensionless form, are given by

(106) $$\frac{\eta_{M}}{2}{\tilde{z}}_{,\tau \tau }=1-{\tilde{\eta }}_{P_{c}},$$

(107) $${\tilde{\eta }}_{P_{c}}(\tau )=\frac{\kappa_{c}}{2}\left(\tilde{w}(0,\tau )-\tilde{z}(\tau )\right)H\left(\tilde{w}(0,\tau )-\tilde{z}(\tau )\right)$$

The solutions of the equations of motion of the beam (16)–(18) with the modification (105) and of the moving mass (106) with (107) can be specified using the time-domain Green functions associated with the beam and the moving mass by

(108) $$\tilde{w}(\xi ,\tau )=8\int_{0}^{\tau }\int_{-\infty }^{\infty }g_{w}(\xi ,\;\varepsilon ,\tau -\varsigma ){\tilde{\eta }}_{P_{c}}(\varsigma )\delta (\varepsilon )d\varepsilon d\varsigma =8\int_{0}^{\tau }g_{w}(\xi ,\;0,\tau -\varsigma ){\tilde{\eta }}_{P_{c}}(\varsigma )d\varsigma ,$$

(109) $$\tilde{z}(\tau )=\int_{0}^{\tau }g_{z}(\tau -\varsigma )\left(1-{\tilde{\eta }}_{P_{c}}(\varsigma )\right)d\varsigma ,$$

where *g_w_*(ξ, ε, τ) is the time-domain Green function of the beam with respect to the moving reference frame, and *g_z_*(τ) is the time-domain Green function of the moving mass. 

The time-domain Green function of the beam in the moving reference frame is obtained by applying the inverse Fourier transform to the frequency-domain Green function of the beam:

(110) $$g_{w}(\xi ,\;\varepsilon ,\tau )=\frac{1}{2\pi }\int_{-\infty }^{\infty }G_{w}(\xi ,\;\varepsilon ,iq)e^{iq\tau }dq,$$

where the frequency-domain Green function of the beam takes the following form:

(111) $$G_{w}(\xi ,\;\varepsilon ,\;iq)=\{\begin{array}{c} G_{w}^{-}(\xi ,\;\varepsilon ,\;iq)=\frac{1}{h_{0}}\sum_{i=1}^{n}\frac{\sum_{j=0}^{4}r_{j}(\bar{q}{)\lambda }_{i}^{4-j}e^{\lambda_{i}(\xi -\varepsilon )}}{\overset{8}{\underset{k=1\;k\neq i}{\Pi }}(\lambda_{k}-\lambda_{i})},\\ G_{w}^{+}(\xi ,\;\varepsilon ,\;iq)=-\frac{1}{h_{0}}\sum_{i=n+1}^{8}\frac{\sum_{j=0}^{4}r_{j}(\bar{q}{)\lambda }_{i}^{4-j}e^{\lambda_{i}(\xi -\varepsilon )}}{\overset{8}{\underset{k=1\;k\neq i}{\Pi }}(\lambda_{k}-\lambda_{i})}. \end{array}$$

However, for ξ = ε = 0, Equation (110) is reduced to

(112) $$g_{w}(\tau )=\frac{1}{2\pi }\int_{-\infty }^{\infty }G_{w}(0,\;0,iq)e^{iq\tau }dq=\frac{1}{2\pi }\frac{1}{h_{0}}\int_{-\infty }^{\infty }\sum_{i=1}^{n}\frac{\sum_{j=0}^{4}r_{j}(\bar{q}{)\lambda }_{i}^{4-j}}{\overset{8}{\underset{k=1\;k\neq i}{\Pi }}(\lambda_{k}-\lambda_{i})}e^{iq\tau }dq,$$

where *g_w_*(τ) = *g_w_*(0, 0, τ). The above integral can be evaluated using a numerical method at any time instant [32,42]. 

The time-domain Green function of the moving mass is

(113) $$g_{z}(\tau )=2\frac{\tau }{\eta_{M}}.$$

Finally, the interaction of the moving mass on the infinite beam on a three-layer viscoelastic foundation is described by Equations (108)–(109) and (106), which can be solved numerically. To this end, the time-domain Green function of the beam, *g_w_*(τ*_n_* – ς), and the dimensionless contact force, ${\tilde{\eta }}_{P_{c}}\left(\varsigma \right)$, are assumed to be linear within the time-step intervals τ*_i_*_–1_ < ς < τ*_i_*:

(114) $$\begin{array}{l} g_{w}(\tau_{n}-\varsigma )=g_{w}(\tau_{n}-\tau_{i-1})+\frac{g_{w}(\tau_{n}-\tau_{i})-g_{w}(\tau_{n}-\tau_{i-1})}{\Delta \tau }(\varsigma -\tau_{i-1}),\\ {\tilde{\eta }}_{P_{c}}\left(\varsigma \right)={\tilde{\eta }}_{P_{c}}{(\tau }_{i-1})+\frac{{\tilde{\eta }}_{P_{c}}{(\tau }_{i})-{\tilde{\eta }}_{P_{c}}{(\tau }_{i-1})}{\Delta \tau }(\varsigma -\tau_{i-1}), \end{array}$$

where Dτ = τ*_i_* − τ*_i_*_−1_. For τ = τ*_n_*, it reads

(115) $${\tilde{w}}_{n}=8\sum_{i=1}^{n}\int_{\tau_{i-1}}^{\tau_{i}}g_{w}(\tau_{n}-\varsigma ){\tilde{\eta }}_{P_{c}}(\varsigma )d\varsigma =8\sum_{i=1}^{n}\frac{1}{2}\left(g_{w,i-1}{\tilde{\eta }}_{P_{c},i}+g_{w,i}{\tilde{\eta }}_{P_{c},i-1}\right)+\frac{1}{3}\left(g_{w,i}-g_{w,i-1}\right)\left({\tilde{\eta }}_{P_{c},i}-{\tilde{\eta }}_{P_{c},i-1}\right),$$

(116) $${\tilde{z}}_{n}=\sum_{i=1}^{n}\int_{\tau_{i-1}}^{\tau_{i}}g_{z}(\tau_{n}-\varsigma )\left(1-{\tilde{\eta }}_{P_{c}}(\varsigma )\right)d\varsigma =\sum_{i=1}^{n}\frac{g_{z,i-1}+g_{z,i}}{2}-\left[\frac{1}{2}\left(g_{z,i-1}{\tilde{\eta }}_{P_{c},i}+g_{z,i}{\tilde{\eta }}_{P_{c},i-1}\right)+\frac{1}{3}\left(g_{z,i}-g_{z,i-1}\right)\left({\tilde{\eta }}_{P_{c},i}-{\tilde{\eta }}_{P_{c},i-1}\right)\right],$$

(117) $${\tilde{\eta }}_{P_{c},n}=\frac{\kappa_{c}}{2}\left({\tilde{w}}_{n}-{\tilde{z}}_{n}\right)H\left({\tilde{w}}_{n}-{\tilde{z}}_{n}\right),$$

where

(118) $$\begin{array}{c} \begin{array}{ccc} {\tilde{w}}_{n}=\tilde{w}(\tau_{n}), & {\tilde{z}}_{n}=\tilde{z}(\tau_{n}), & {\tilde{\eta }}_{P_{c},n}= \end{array}{\tilde{\eta }}_{P_{c}}(\tau_{n})\\ \begin{array}{cc} g_{w,i}=g_{w}(\tau_{n}-\tau_{i}), & g_{z,i}=g_{z}(\tau_{n}-\tau_{i}) \end{array}. \end{array}$$

By introducing Equations (115) and (116) into Equation (117), a linear equation for ${\tilde{\eta }}_{P_{c},n}$ is obtained, and the dimensionless contact force at time τ*_n_* is given as its solution. The dimensionless displacement of the beam and of the moving mass can then be calculated using Equations (115) and (116).

## 5. Numerical Application

### 5.1. Allowable Intervals of Dimensionless Parameters

In this section, the allowable ranges of dimensionless parameters are identified. To accomplish this, one must first know the reasonable ranges of possible real values. As for the rail, the range of possible values is quite narrow; basically, there are only two guide data sets for 54 E1 and 60 E1, specifying values of *EI* and *m*. The sleepers can be wooden or concrete, and therefore, their mass varies from approximately 80 kg to 320 kg [71]. Since only one rail is considered, only half the sleeper mass is introduced. Admitting sleeper spacings from 0.545m [73] to 0.711 m [74], the distributed masses for half sleepers can be determined. There are studies on larger sleeper spacings [75] that could provide significant cost savings; nevertheless, these values are not yet common in practice and will therefore not be considered. As for the limit values for *k_f_*, they are taken from [76,77], respectively.

As for the ballast contribution, the stress cone (prism) theory can be used to estimate some realistic values [72,73]. Admitting the effective sleeper length according to [71] and then varying the sleepers’ spacing from 0.545 to 0.711 m, the sleepers’ base from 0.22 to 0.35 m, the ballast height from 0.2 to 0.6 m, and the angle of distribution from 25° to 50°, the formulas from [72] indicate the stiffness coefficient range 0.847–3.261 m and the volume of dynamically activated mass coefficient range 0.069–0.630 m^3^. Additionally, the shear contribution coefficient range is 0.03–0.5 m. Admitting the ballast Young’s modulus from 50 MPa to 400 MPa, the ballast density within 1200–2600 kg/m^3^, and the Poisson ratio within 0.1–0.45, real values can be determined and then distributed according to the sleeper spacing. All of these values are summarized in Table 1.

The admissible ranges of the dimensionless parameters are summarized in Table 2. These ranges are extended by a certain margin, since all considered real values are not exact limits. η*_s_* is sometimes omitted in similar models so that the lower bound is allowed to be zero and only the upper bound is estimated. Regarding the damping coefficients, very different values can be found in the literature, so it was decided to directly vary the dimensionless parameters. 

As for η*_M_*, academic values will be used here to explain in detail all the tendencies affecting the onset of instability. In general, there are studies that associate mass with force as its weight, and then the mass could be as high as 10 t, but if only the wheel mass is considered, then it can be as low as 880 kg. Additionally, there are also intermediate approaches that add a corresponding axle mass or part of the boogie mass to the wheel mass. According to Table 1, the value of χ varies between 0.3 and 2.7 m^−1^, giving the range of η*_M_* as 50–500 for 10 t and 4.4–44 for 880 kg.

In addition to studies with parameters selected from the allowable ranges, one specific case of a real railway is also considered. This case is taken from [73]. Using the real values from [73], the dimensionless parameters are calculated as

μ*_s_* = 3.8, μ*_b_* = 16, κ*_p_* = 0.8, κ*_b_* = 1.8, η*_s_* = 0.7, η*_p_* = 2.2 ∙ 10^−3^, η*_b_* = 1.8 ∙ 10^−3^, η*_f_* = 0.9 ∙ 10^−3^, (119)

### 5.2. Test Case from [73]

**Figure 5:**
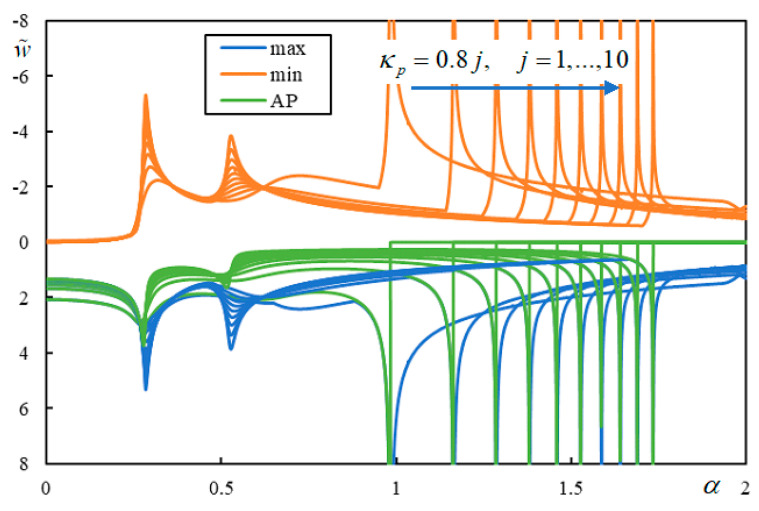
Parametric analysis related to μ*_s_* = 3.8, μ*_b_* = 16, κ*_p_* = 0.8*j*, *j* = 1, …, 10, κ*_b_* = 1.8, η*_s_* = 0.7, and η*_p_* = η*_b_* = η*_f_* = 0 (max/min—global maximum/minimum deflection over the whole beam; AP—deflection at the active point).

In this section, a real case of the railway track from [73] is selected for analysis. First, the critical velocity of the moving force is determined because it provides important data for further study. According to Section 3.3, it can be shown that this case has only one resonance. A valid pair for this is $\left(p,\alpha_{cr}^{st}\right)=\left(1.456,\;0.983\right)$, which can also be confirmed by the analysis described in Section 3.4. Parametric analysis reveals that there are two non-dominant pseudocritical velocities (PCV1 and PCV2) lower than the critical one (CV). Nevertheless, by gradually increasing κ*_p_* from 0.8 up to 8, which can be easily achieved by increasing the stiffness of the rail pads, still in a range related to soft rubber, it can be seen that while CV is increasing, the PCVs are gaining its importance. The results of these parametric analyses are summarized in Figure 5. 

**Figure 6:**
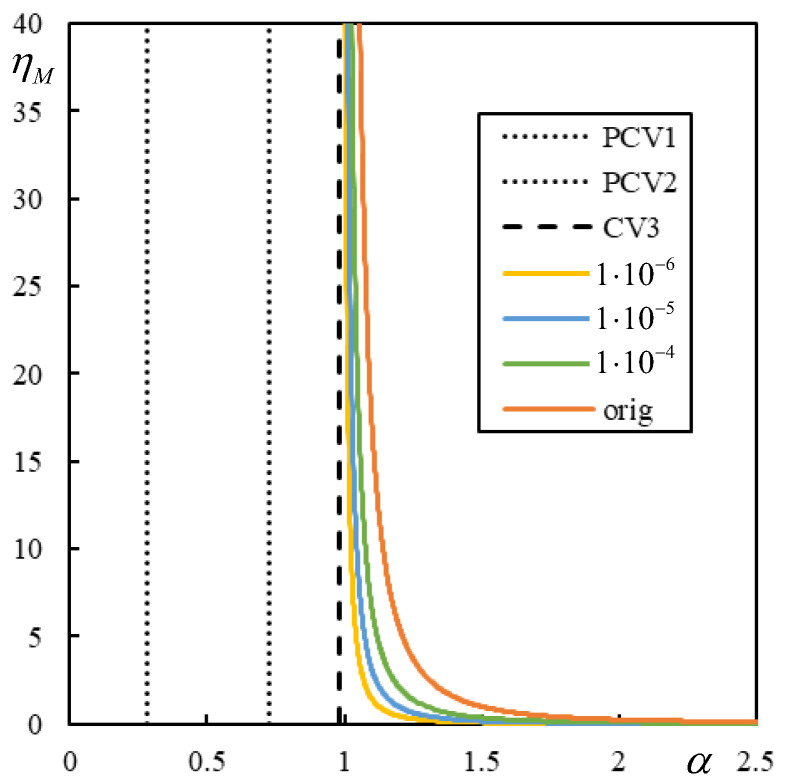
Instability lines related to μ*_s_* = 3.8, μ*_b_* = 16, κ*_p_* = 0.8*j*, *j* = 1, …, 10, κ*_b_* = 1.8, and η*_s_* = 0.7 (original damping parameters η*_p_* = 2.2 · 10^−3^, η*_b_* = 1.8 · 10^−3^, η*_f_* = 0.9 · 10^−3^; 1 · 10^−4^, 1 · 10^−5^, 1 · 10^−6^—equal damping for η*_p_* = η*_b_* = η*_f_*).

Further investigation shows how these values affect the instability lines. Even if the CVs and PCVs are different for different j, there are basically three distinct regions where the instability branches are contained. For the original value κ*_p_* = 0.8, the PCVs are not dominant, and therefore, there is only one instability branch, as shown in Figure 6. 

**Figure 7:**
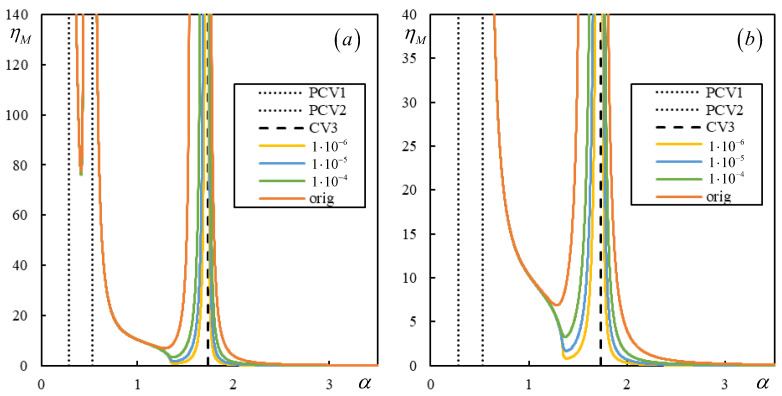
Instability lines related to μ*_s_* = 3.8, μ*_b_* = 16, κ*_p_* = 8, κ*_b_* = 1.8, and η*_s_* = 0.7 (original damping parameters η*_p_* = 2.2 ∙ 10^−3^, η*_b_* = 1.8 ∙ 10^−3^, η*_f_* = 0.9 ∙ 10^−3^; 1 ∙ 10^−4^, 1 ∙ 10^−5^, 1 ∙ 10^−6^—equal damping for η*_p_* = η*_b_* = η*_f_*; (**a**,**b**) correspond to different η*_M_*-scales).

**Figure 8:**
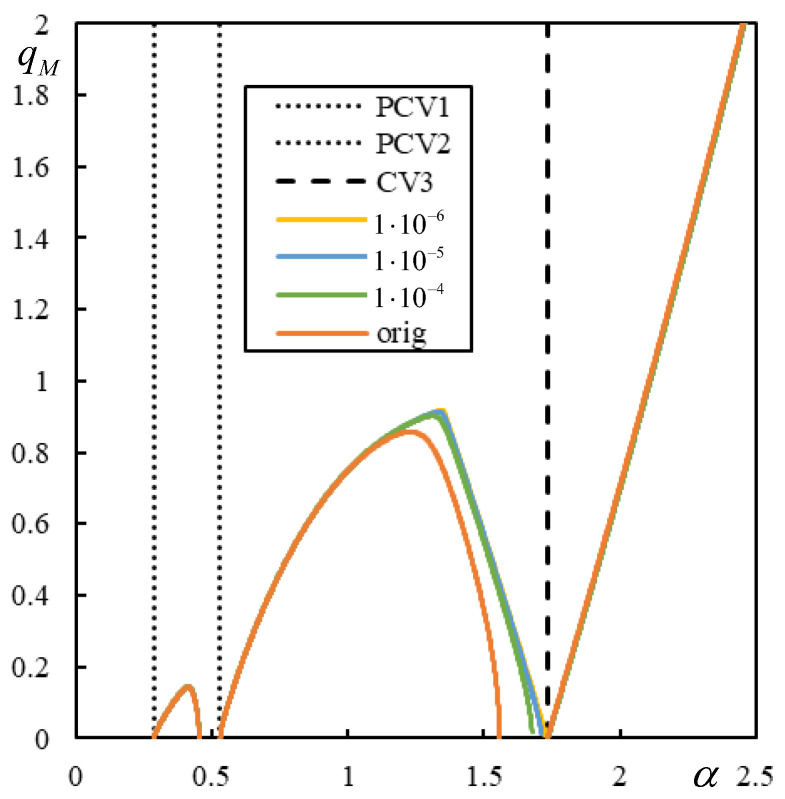
Real-valued poles *q_M_* related to μ*_s_* = 3.8, μ*_b_* = 16, κ*_p_* = 8, κ*_b_* = 1.8, and η*_s_* = 0.7 (original damping parameters η*_p_* = 2.2 ∙ 10^−3^, η*_b_* = 1.8 ∙ 10^−3^, η*_f_* = 0.9 ∙ 10^−3^; 1 ∙ 10^−4^, 1 ∙ 10^−5^, 1 ∙ 10^−6^—equal damping for η*_p_* = η*_b_* = η*_f_*).

For the highest value tested, κ*_p_* = 8, the branches of the instability lines are contained in all three regions and do not cross the CV or PCVs, as demonstrated in Figure 7. For completeness, the corresponding real-valued *q_M_* values are also plotted in Figure 8. 

A detailed analysis shows that there is a branch in the first region already from *j* = 2 and in the second region from *j* = 3. This is important from an analytical point of view; from a practical point of view, it is also necessary to evaluate whether the η*_M_* values in these branches are feasible. 

### 5.3. Other Test Cases

In this section, results related to several other cases are presented. It is concluded that they can be classified as *regular*, i.e., with expected behavior, and *irregular*. The regular cases behave as in Section 5.2, meaning that the following apply:

(i) There is at most one instability branch in each of the three regions delimited by CVs and PCVs;
(ii) No branch intersects CV or PCV;
(iii) Branches that correspond to lower damping are below the ones with higher damping, and they do not cross;
(iv) In the first two regions, the branches asymptotically tend to infinite η*_M_*, and in the last region, they asymptotically tend to infinite η*_M_* from the left and zero η*_M_* from the right.

This regularity is not associated with the number of resonances. Irregular cases then violate one of these properties, especially at low damping levels.

Cases selected for further analysis are listed in Table 3, in which “nd” and “d” designate dominant and non-dominant, respectively.

**Figure 9:**
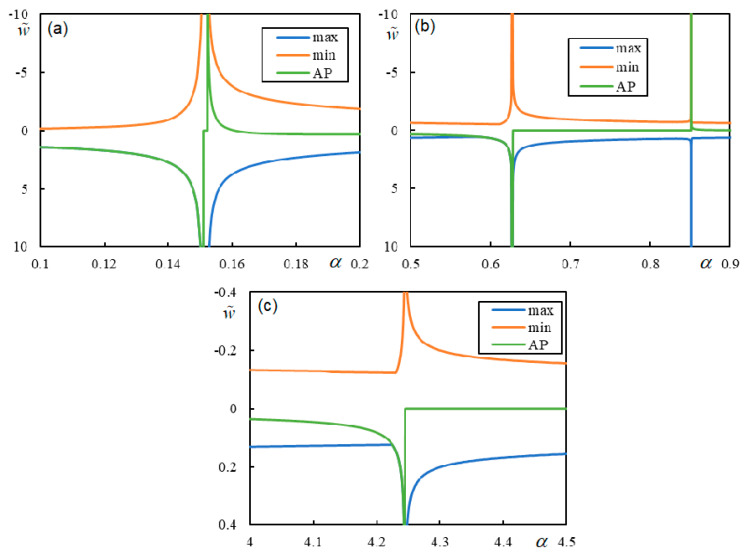
Parametric analysis related to Case 1 (max/min—global maximum/minimum deflection over the whole beam; AP—deflection at the active point; (**a**–**c**) correspond to different α-scales).

**Figure 10:**
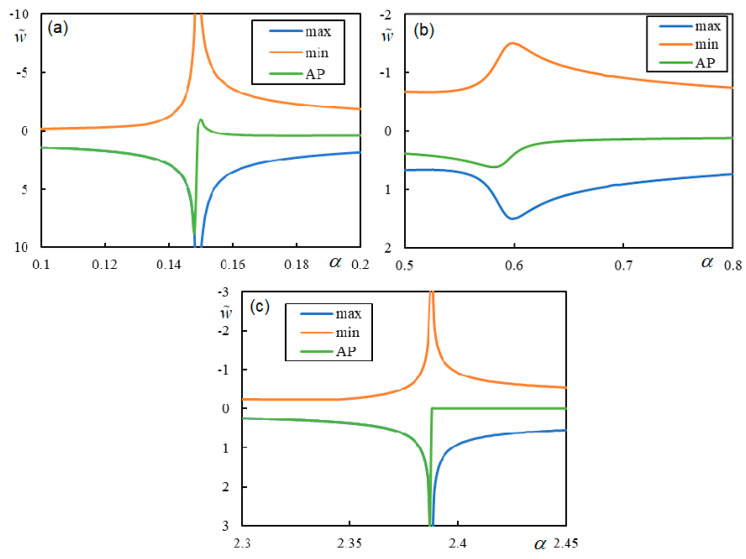
Parametric analysis related to Case 2 (max/min—global maximum/minimum deflection over the whole beam; AP—deflection at the active point; (**a**–**c**) correspond to different α-scales).

**Figure 11:**
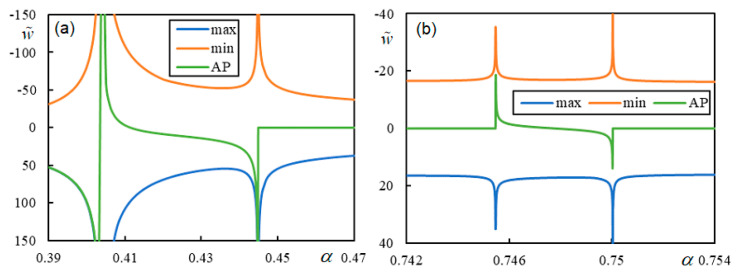
Parametric analysis related to Case 3 (max/min—global maximum/minimum deflection over the whole beam; AP—deflection at the active point; (**a**,**b**) correspond to different α-scales).

**Figure 12:**
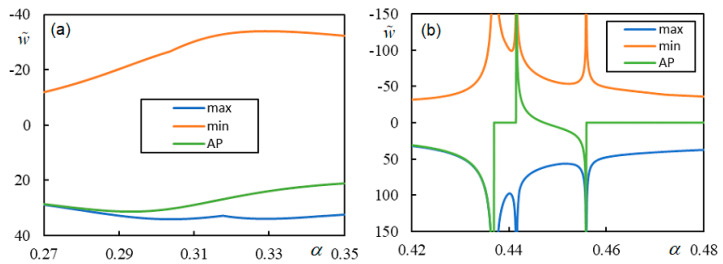
Parametric analysis related to Case 4 (max/min—global maximum/minimum deflection over the whole beam; AP—deflection at the active point; (**a**,**b**) correspond to different α-scales).

First, the effect of the number of resonances is demonstrated in Figure 9, Figure 10, Figure 11 and Figure 12 in the graphs obtained by parametric analysis. The following observations can be derived from Figure 9, Figure 10, Figure 11 and Figure 12. 

In Case 1 (Figure 9), all five resonances are clearly marked. The first two are quite close; however, the sudden jump to zero deflection at the active point for velocities between CV1 and PCV is undoubtedly noticeable. 

In Case 2 (Figure 10), PCV1 is dominant, but PCV2 is not. Nevertheless, both are ambiguous because, for an α-step of 0.001, the extreme values on the entire beam are achieved at the same α reported in Table 3, but the extreme values at the active point occur at 0.148 and 0.581, respectively. 

In Case 3 (Figure 11), PCV1 is dominant, and the extreme values are reached at the same α with an accuracy of 0.001, while in Case 4 (Figure 12), PCV1 is non-dominant and highly ambiguous because the extreme values are achieved for the maximum at 0.3037 and 0.3291 and for the minimum at 0.3288 and at the active point at 0.2932 with an accuracy of 0.0001. In particular, Case 2 shows that CV cannot be determined only analytically, as this could lead to the incorrect conclusion that instability can only occur for α > 2.388. This means that when the number of resonances is less than five, an additional parametric analysis must be performed. However, once the PCVs are determined, it is difficult to discern their dominance, i.e., their direct influence on the instability lines.

**Figure 13:**
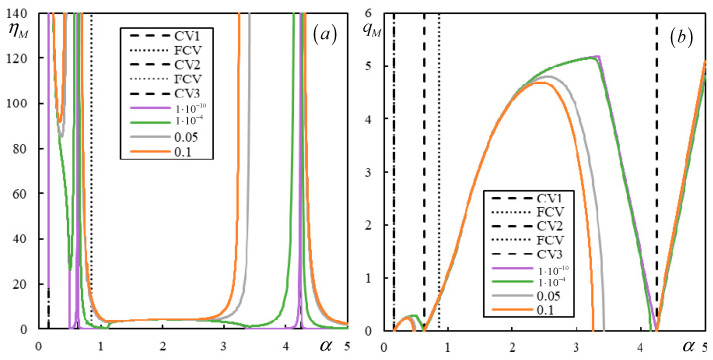
Case 1: (**a**) instability lines; (**b**) associated real-valued *q_M_* (resonance values according to Table 3; 0.1, 0.05, 1 ∙ 10^−4^, 1 ∙ 10^−10^—equal damping for η*_p_* = η*_b_* = η*_f_*).

**Figure 14:**
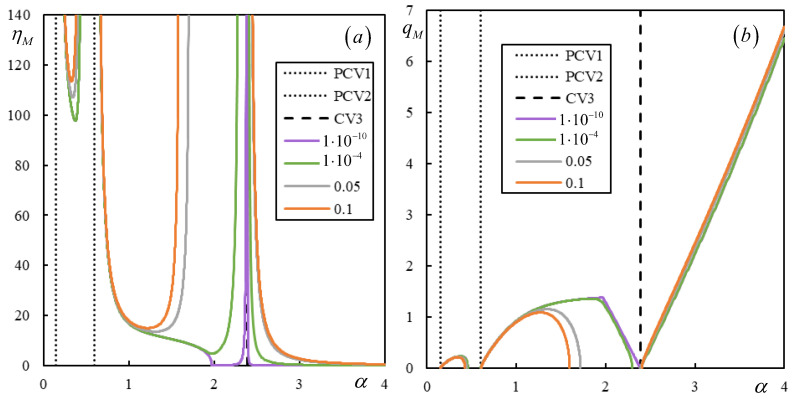
Case 2: (**a**) instability lines; (**b**) associated real-valued *q_M_* (resonance values according to Table 3; 0.1, 0.05, 1 ∙ 10^−4^, 1 ∙ 10^−10^—equal damping for η*_p_* = η*_b_* = η*_f_*).

Further analysis will show the instability lines and their connection to the CVs in both regular and irregular cases. Cases 1 and 2 are visualized in Figure 13 and Figure 14. In Figure 13 and Figure 14, features of the regular behavior that is consistent with the one- and two-layer models can be observed: (i) the instability branches are contained in the three regions delimited by the vertical lines of CV or PCV; (ii) the instability lines for lower damping levels are below the lines for higher damping, and they do not cross; (iii) there is only one instability branch in each region; (iv) with decreasing damping, the instability lines approach the CV or PCV faster from the left than from the right; and (v) instability branches tend asymptotically to infinite η*_M_* because *q_M_* is tending to zero, except for the last region, where the right-hand end tends to zero η*_M_* for infinite α. In addition, the first two regions can be left without any branches, as in Figure 6. Furthermore, FCV has no effect on the instability lines, but this property is preserved even in irregular cases. In Case 1, there is only one exception for very low damping. The instability line for η*_p_* = η*_b_* = η*_f_* = 1 × 10^−10^ adopts a strange shape in the first region, which will be discussed in the next section. 

**Figure 15:**
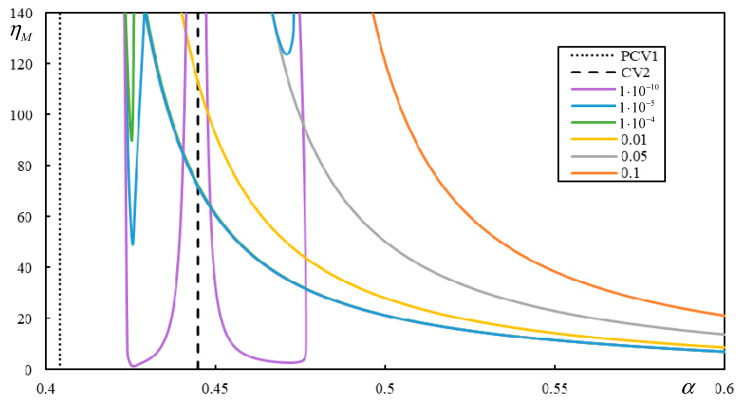
Instability lines in Case 3 (resonance values according to Table 3; 0.1, 0.05, 0.01, 1 ∙ 10^−4^, 1 ∙ 10^−10^—equal damping for η*_p_* = η*_b_* = η*_f_*).

**Figure 16:**
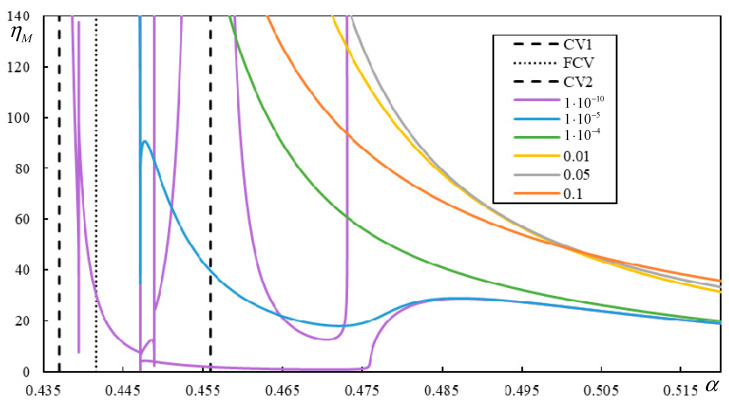
Instability lines in Case 4 (resonance values according to Table 3; 0.1, 0.05, 0.01, 1 ∙ 10^−4^, 1 ∙ 10^−10^—equal damping for η*_p_* = η*_b_* = η*_f_*).

Further, Cases 3 and 4 are irregular, which means that properties (i)–(v) are not preserved. This is demonstrated in Figure 15 and Figure 16. 

In Figure 15, the α-scale is not extended up to CV3 for better clarity. On the other hand, for the same reason, the α-scale does not cover the non-dominant PCV in Figure 16. In both cases, the instability branches can be seen to intersect CV and cross each other, and at low damping levels, strange shapes and additional branches appear. Here, in regard to (v), the instability lines can tend to infinite η*_M_* due to *K*(0, *q*) tending to zero, which is a new feature.

In summary, by fixing a particular moving mass ratio, meaning by tracing a horizontal line in the graphs of Figure 6, Figure 7a, Figure 13a, Figure 14a, Figure 15 and Figure 16, the dependence of the critical velocity on the damping level is demonstrated. It is seen that in regular cases, there is a smooth increase in the critical velocity with the damping level; however, this dependence can be suddenly interrupted for a certain damping value or changed in irregular cases. Some of the irregularities will be discussed further in the next section. 

### 5.4. Influence of the Damping of the Materials in the Foundation

In this section, the effect of damping in foundation materials is discussed. This issue is very sensitive to the critical velocity of a moving load [78,79]. The critical velocity of one moving mass in the undamped case is expected to match the critical velocities of the moving force. Therefore, the instability lines for very low damping values should be close to the vertical lines indicating CVs or PCVs. However, it has been observed that very low damping values can lead to rather peculiar forms of the instability branches. Additionally, there are more branches than predicted by the number of regions, and some of the new branches can even be closed. 

**Figure 17:**
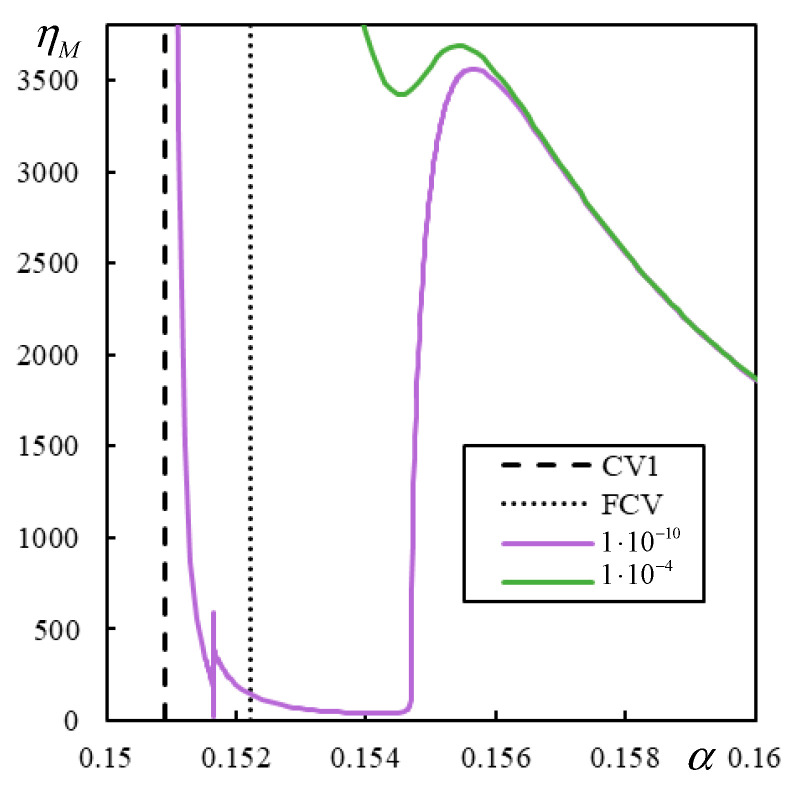
Instability lines in Case 1 (resonance values according to Table 3; 1 ∙ 10^−4^, 1 ∙ 10^−10^—equal damping for η*_p_* = η*_b_* = η*_f_*).

Figure 17 shows the initial part of the first branch for two damping levels in Case 1. The η*_M_*-scale in Figure 17 is extended to academic values to demonstrate that even though the properties of the regular behavior are preserved, there is no consistency in the sense that the critical velocity of the moving mass varies smoothly as the damping decreases. 

Figure 17 shows that for realistic η*_M_*, there is a sudden jump, and two more closed intervals of instability appear at a specific damping value, covering α-values significantly lower than the previous ones. Although the exponential growth of unstable behavior is very slow in such cases, it will lead to instability. The presented results are calculated with very high precision achieved by a high number of significant digits, which is possible in symbolic software. 

**Figure 18:**
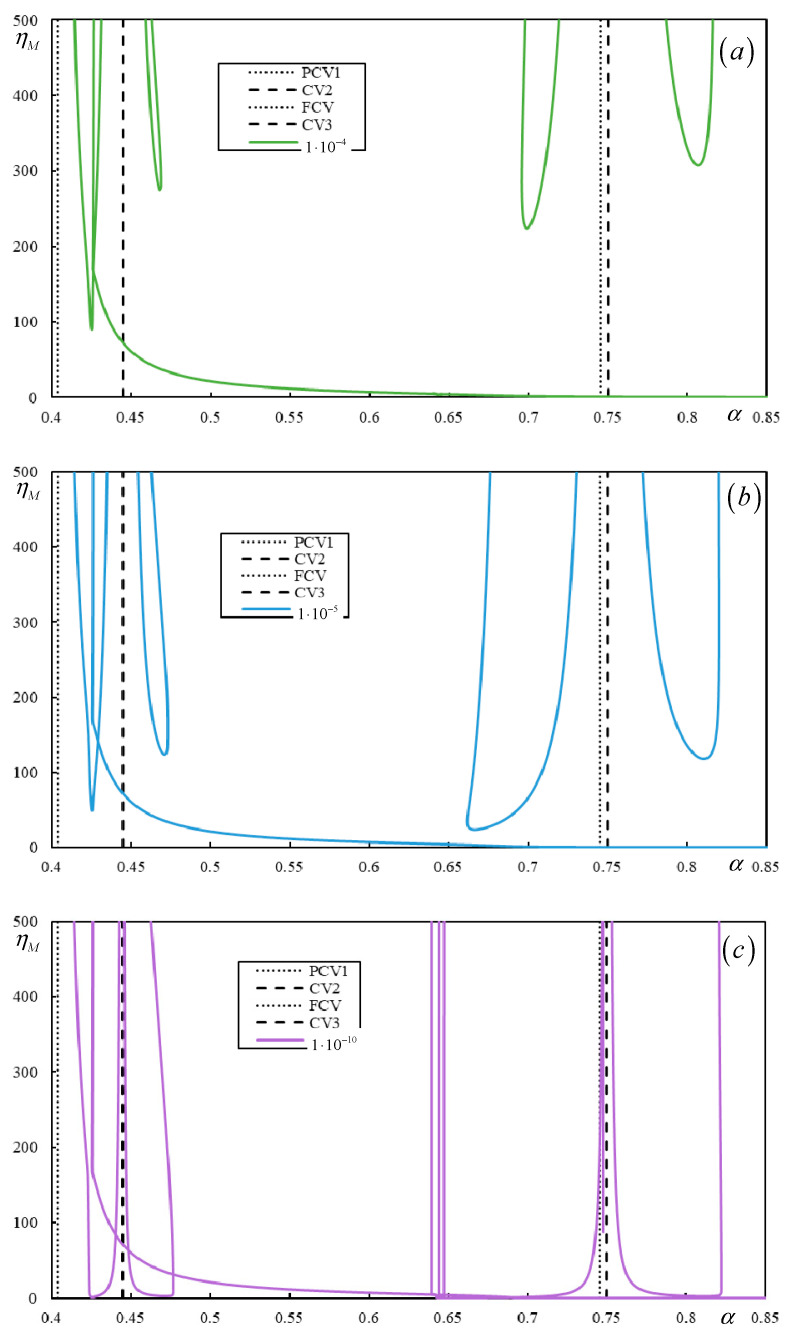
Instability lines in Case 3: (**a**) η*_p_* = η*_b_* = η*_f_* = 1 × 10^−4^; (**b**) η*_p_* = η*_b_* = η*_f_* = 1 ∙ 10^−5^; (**c**) η*_p_* = η*_b_* = η*_f_* = 1 ∙ 10^−10^ (resonance values according to Table 3).

**Figure 19:**
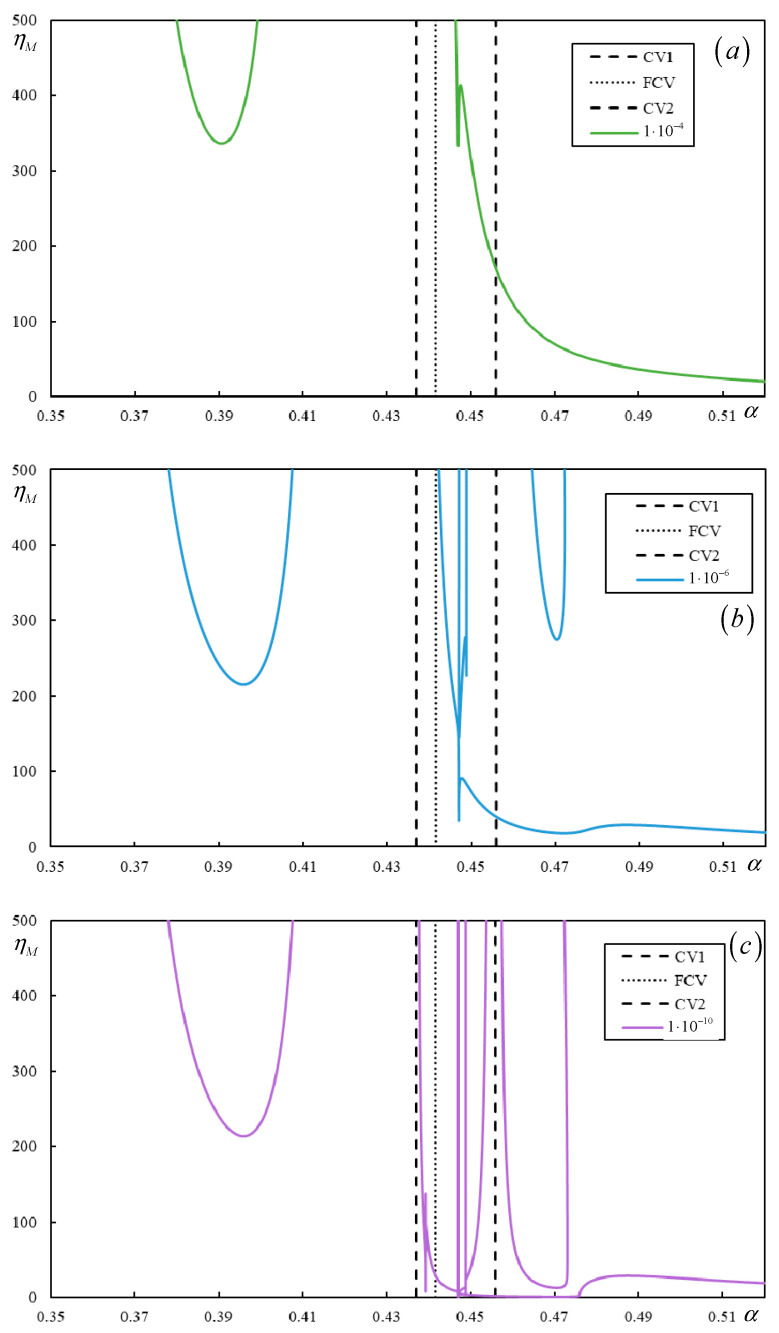
Instability lines in Case 4: (**a**) η*_p_* = η*_b_* = η*_f_* = 1 ∙ 10^−4^; (**b**) η*_p_* = η*_b_* = η*_f_* = 1 ∙ 10^−6^; (**c**) η*_p_* = η*_b_* = η*_f_* = 1 ∙ 10^−10^ (resonance values according to Table 3).

Figure 18 and Figure 19 show the instability branches for low damping levels for Cases 3 and 4, again with the η*_M_*-scale extended to academic values. 

It can be observed in Figure 18 that, especially at low damping levels, the instability lines have rather complicated shapes. There are six, six, and seven branches for η*_p_* = η*_b_* = η*_f_* = 1 ∙ 10^−4^, η*_p_* = η*_b_* = η*_f_* = 1 ∙ 10^−5^, and η*_p_* = η*_b_* = η*_f_* = 1 ∙ 10^−10^, respectively. Each mentioned case has one of the branches closed. For lower damping, these branches attain realistic values of η*_M_*. 

In Figure 19, it is possible to observe how the number of instability branches increases with decreasing damping. Although the η*_M_*-scale is academic, there are other branches not covered by the scale. In Figure 19, PCV1, which was identified as non-dominant and highly ambiguous (between 0.2932 and 0.3291), is not plotted, but it can be seen that it serves to delimit the first region where instability branches appear. However, instability is unrealistic in this case due to very high η*_M_*. 

There are five, six, and six branches for η*_p_* = η*_b_* = η*_f_* = 1 ∙ 10^−4^, η*_p_* = η*_b_* = η*_f_* = 1 ∙ 10^−6^, and η*_p_* = η*_b_* = η*_f_* = 1 × 10^−10^, respectively, and for lower damping, these branches reach realistic values of η*_M_*. Here, one of the branches is closed only for η*_p_* = η*_b_* = η*_f_* = 1 ∙ 10^−6^ and η*_p_* = η*_b_* = η*_f_* = 1 ∙ 10^−10^. 

### 5.5. Comparison between the Results Obtained Using the Semi-Analytical Method and Green’s Function Method

In this section, some results regarding the critical velocity of the moving mass and its time evolution obtained using the semi-analytical method and the method based on the Green’s function are presented. In fact, several of the results presented in the previous sections have been verified using the D-decomposition method based on the Green’s function, and to avoid redundant presentation, only one case is presented here.

**Figure 20:**
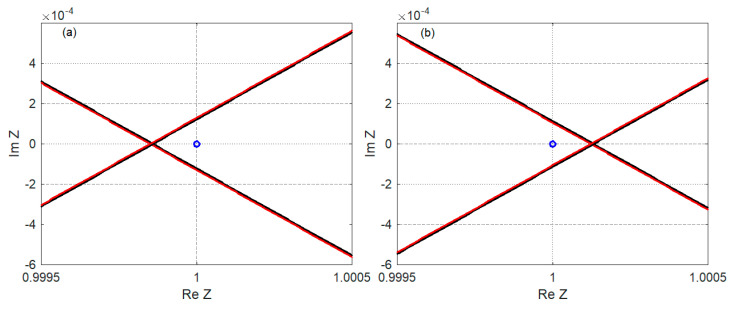
D-decomposition for Case 3 (η*_p_* = η*_b_* = η*_f_* = 0.01 and η*_M_* = 60): (**a**) α = 0.46369 -stable; (**b**) α = 0.46370 − unstable; —, (Re *Z*(α, ±i*q*), Im *Z*(α, ±i*q*)); —, (Re Z(α, ζ ± i*q*), Im Z(α, ζ ± i*q*)), ⸰, point of coordinates (Re *Z* = 1, Im *Z* = 0).

Figure 20 shows the D-decomposition diagrams determining the critical velocity in Case 3 for η*_p_* = η*_b_* = η*_f_* = 0.01 and η*_M_* = 60. When the moving mass velocity is 0.46369, the system is stable, and when the velocity is 0.46370, the system is unstable, meaning that the critical velocity is between these two values. This result corresponds to the one displayed in Figure 15 (yellow curve). 

When calculating the time series of the moving mass and the beam using the algorithm based on the Green’s function presented in Section 4.2, the result depends on the stiffness of the elastic element inserted between the moving mass and the beam. 

**Figure 21:**
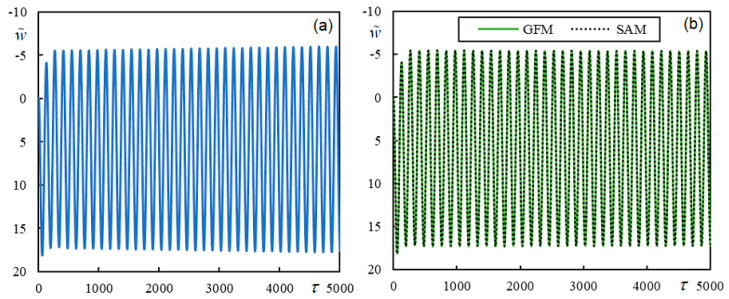
Time series of the beam at the moving point for (**a**) Green’s function method with κ*_c_* = 94.25049; (**b**) Green’s function method with κ*_c_* = 9425.049 (GFM) and the semi-analytical approach (SAM).

Figure 21 shows the time series of the beam at the moving contact point at a dimensionless velocity of 0.46369, considering two values for the dimensionless stiffness of the elastic element, namely, κ*_c_* = 94.25049 and κ*_c_* = 9425.049. The calculation was performed in the MATLAB environment using a laptop with an Intel Core i5-8265U CPU @ 1.60 GHz 1.80 GHz microprocessor and installed RAM of 8.00 GB. The calculation time is approximately 7850 s, of which 6610 s is for the calculation of the Green’s function and the remaining 1240 s is for the calculation of the convolution integral with the determination of the quantities of interest: the displacement of the moving mass and the beam and the contact force. The time step used was Δτ = 0.0258. 

When the dimensionless stiffness of the moving mass/beam contact is low (Figure 21a), the system is unstable, but for sufficiently high dimensionless stiffness, the time series shows that the system is stable (Figure 21b). In the semi-analytical approach, fixed contact is assumed; therefore, in this case, the vibrations induced by the moving mass are stable. These results are included in Figure 21b, which shows that the two curves overlap. For a numerical evaluation using the semi-analytical approach, it is necessary to make several choices. First, given that *K*(0, *q*), like the full function to be integrated into Equation (41), has a symmetric real part and an antisymmetric imaginary part when *q* ∈ (−i*a* − ∞, −i*a* + ∞), and by exploiting Euler’s formula with *q* = −i*a* + *q_r_*, the following holds:

(120) w˜(0,τ)=1π∫−ia−∞−ia+∞−2iηCK(0,q)q(π−2ηMq2K(0,q))eiqτdq=∫0∞2eaτ(Re(−2iηCK(0,q)qπ(π−2ηMq2K(0,q)))cos(qrτ)−Im(−2iηCK(0,q)qπ(π−2ηMq2K(0,q)))sin(qrτ))dqr.

It is clear from Equation (120) that *a*, which is positive, must be chosen not only with respect to the definition of the inverse Laplace transform but also with respect to the time interval that is considered for the evaluation. When performing calculations in MATLAB, the numerical precision is compromised at very high τ due to the inherent double precision of this software. Unfortunately, the numerical evaluation of two embedded integrals with exact infinite limits is extremely time-consuming and does not allow any simplification. Therefore, it is best to use a simple trapezoidal rule for the evaluation of the integral in Equation (120). To this end, all values not involving τ can be prepared and then used with any chosen τ. This means that no previous time results are necessary, and thus, the only criterion for the time step is the smoothness of the resulting curve. In this case, Δτ = 5 was found to be sufficient. In this approach, the calculation time is dependent on the number of values used in the trapezoidal rule, because after that, the evaluation is practically instantaneous. 

When performing calculations in MATLAB, it is more convenient to calculate the *K*-function values numerically using a predefined MATLAB integration subroutine. This is because, for contour integration, double precision could compromise the pole and residue evaluation. The calculation time is practically the same if the *p*-scale is extended to infinity or some reasonable value. Therefore, the most important choices concern the scale and the step of *q_r_*. Generally, the smaller the *a*-value, the larger the concentration of the function to be integrated around *q_r_* = 0, i.e., around *q* = −i*a*. This allows for a shorter *q_r_*-interval but requires a lower *q_r_*-step. After calculating some values, their graph can indicate whether the choices are adequate or not. Numerically, each choice should be confirmed by convergence analysis. One of the criteria to be met, in addition to being independent of refined choices, is a zero value at zero time. For the graph in Figure 21b, *a =* 0.0001, *q_r_*-step= 5 × 10^−5^, and *q_r_* limited by 2.5 were chosen, which involved the evaluation of 50,000 values using the trapezoidal rule and took 947 s on a laptop with the same characteristics as specified before. 

To evaluate the accuracy of the results given in Figure 21b, the RMS value of the difference between the two graphs was evaluated using discrete values with a time step of Δτ = 5, that is, using 1001 values, and the result was 0.44%, which is very good.

**Figure 22:**
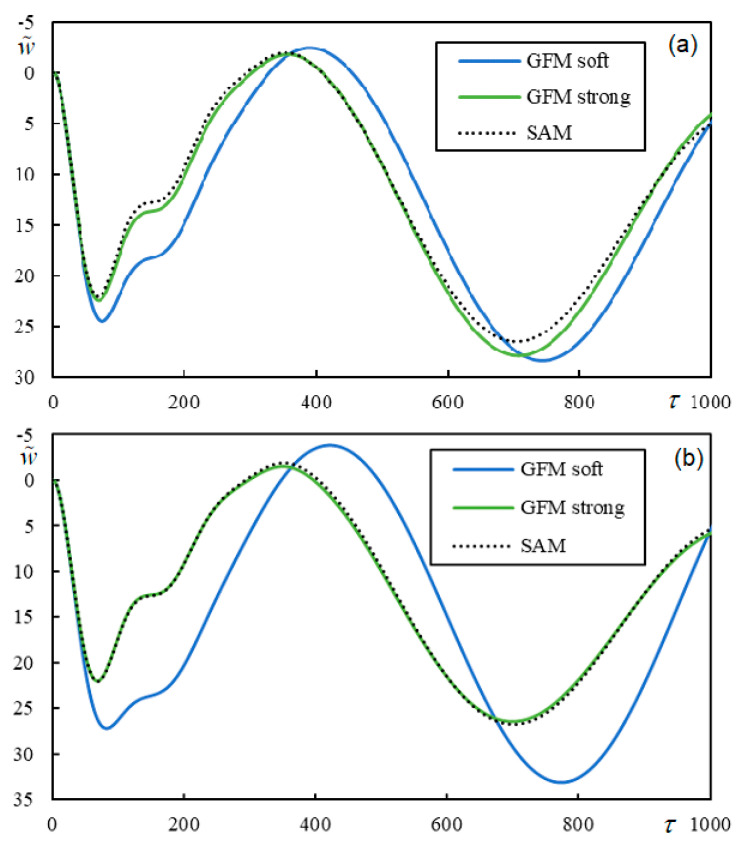
Time series of the beam at the moving point: Green’s function method with κ*_c_* = 375 (GFM soft) and with κ*_c_* = 23,563 (GFM strong) and semi-analytical approach (SAM): (**a**) α = 0.4157; (**b**) α = 0.4158.

To conclude, Case 4, with a low level of damping η*_p_* = η*_b_* = η*_f_* = 1 × 10^−6^, a moving mass ratio η*_M_* = 60, and two values of the velocity ratio, α = 0.4517 and α = 0.4518, is selected to demonstrate how the results obtained by the Green’s function method approach those obtained by the semi-analytical method by increasing the stiffness of the contact spring. The results are presented in Figure 22. 

α = 0.4517 belongs to the stable interval, and α = 0.4518 belongs to the unstable one, with the exact separation being α = 0.451710491628607, but since the degree of instability is very low, the difference would only be seen after a very long time. As expected, by increasing the contact stiffness, the contact becomes closer to rigid, and the results of both methods are identical.

## 6. Conclusions

In this paper, a detailed analysis of an infinite beam on a three-layer viscoelastic foundation subjected to a moving mass is presented. Three main objectives are listed: (i) the identification of the lowest velocity at the stability limit; (ii) the analysis of the damping influence of the foundation materials; and (iii) a comparison of the methods implemented in this paper, namely, the semi-analytical approach and the approach based on the Green’s function method.

Using the Green’s function method to calculate the time series of a moving mass on an infinite beam on a three-layer foundation requires elastic contact between the moving mass and the beam and the presence of damping in the foundation materials to ensure numerical stability. The advantage of this method is the possibility of taking into account the nonlinearity of the contact between the moving mass and the beam, namely, the nonlinear character of the elastic contact and the possibility of contact loss [32,40,42]. On the other hand, the D-decomposition method developed in terms of the Green’s function method can be applied to calculate the critical velocity of the moving mass on a beam on a three-layer foundation with high accuracy when the foundation is damped. 

The main advantages of the semi-analytical approach are listed as follows: (i) there are no numerical problems in cases without any damping; (ii) there are no numerical issues in considering a rigid contact (however, a linear contact spring can also be included); and (iii) many related results, such as the critical velocity of the moving force, the analysis on finite beams, etc., are presented in analytical form. There are just no limitations; the results are highly accurate, and the formulations developed in this paper can be easily used by other researchers. Unfortunately, the main advantage of the new approach ([10,34,35,38,59]), which is so effective in one- and two-layer models, is lost in the three-layer model, because the mass-induced frequencies are not well defined in most cases, and therefore, the analytical expression for the unsteady harmonic part cannot be used in such cases. A regularization approach to overcome this difficulty is currently in preparation. This is why the time series in this paper are presented with the numerical evaluation of the inverse Laplace transform. However, the accuracy and calculation times have been shown to be very similar to the Green’s function method. In summary, time series in simpler models, like one- or two-layer models, were much simpler and more advantageous because it was possible to identify the so-called unsteady harmonic part, which was determined analytically, and thus, the numerical evaluation was practically instantaneous and could be extended to infinite time without loss of accuracy. However, other advantages of the semi-analytical approach related to the determination of instability lines are preserved for all layered models, and these lines can be determined very quickly and accurately while keeping the main part of the calculations in the real domain.

## Figures and Tables

**Table 1:** Ranges of typical values of the main parts of the three-layer model.

Parameter	Approximate Range (with Margins)
*EI* (MNm^2^)	4.7 or 6.4
*m* (kg/m)	54 or 60
*m_s_* (kg/m)	56–294
*m_b_* (kg/m)	117–2377
*k_p_* (MN/m^2^)	28–9174
*k_b_* (MN/m^2^)	42–1304
*k_f_* (MN/m^2^)	0.22–1000
*k_s_* (MN/m^2^)	0.5–141

**Table 2:** Range of some dimensionless values of the main parts of the three-layer model.

Dimensionless Parameter	Approximate Range (with Margins)
μ*_s_*	1–6
μ*_b_*	2–45
κ*_p_*	0.03–42,000
κ*_b_*	0.04–6000
η*_s_*	0–70

**Table 3:** Other selected cases to exemplify the influence of the resonances and the classification.

Case	μ*_s_*	μ*_b_*	κ*_p_*	κ*_b_*	η*_s_*	Resonances	Type
1	6	35	300	7	0	5 (0.151-CV1; 0.152-FCV; 0.627-CV2; 0.851-FCV; 4.244-CV3)	regular
2	6	35	30	7	0	1 (0.149-PCV1 (nd); 0.599-PCV2; 2.388-CV3))	regular
3	6	5	0.03	3	0	3 (0.404-PCV1 (d); 0.445-CV2; 0.745-FCV; 0.750-CV3)	irregular
4	3	10	0.03	0.1	0	3 (0.293-PCV1 (nd); 0.437-CV2; 0.442-FCV; 0.456-CV3)	irregular

## Data Availability

Data are contained within the article.

## Appendix A

The $h_{j}(\alpha ,\bar{q})$ coefficients from Equation (87) can be calculated as follows:

(A1) $$h_{0}=c_{10}c_{20}c_{30}$$

(A2) $$h_{1}=c_{10}c_{20}c_{31}+c_{10}c_{21}c_{30}$$

(A3) $$h_{2}=c_{10}c_{20}c_{32}+c_{10}c_{21}c_{31}+c_{10}c_{22}c_{30}+c_{12}c_{20}c_{30}-c_{10}c_{60}^{2}$$

(A4) $$h_{3}=c_{10}c_{21}c_{32}+c_{10}c_{22}c_{31}+c_{12}c_{20}c_{31}+c_{12}c_{21}c_{30}+c_{13}c_{20}c_{30}-2c_{10}c_{60}c_{61}$$

(A5) $$\begin{array}{l} h_{4}=c_{10}c_{22}c_{32}+c_{12}c_{20}c_{32}+c_{12}c_{21}c_{31}+c_{12}c_{22}c_{30}+c_{13}c_{20}c_{31}+c_{13}c_{21}c_{30}+c_{14}c_{20}c_{30}-\\ c_{30}c_{40}c_{50}-c_{10}c_{61}^{2}-c_{12}c_{60}^{2} \end{array}$$

(A6) $$\begin{array}{l} h_{5}=c_{12}c_{21}c_{32}+c_{12}c_{22}c_{31}+c_{13}c_{20}c_{32}+c_{13}c_{21}c_{31}+c_{13}c_{22}c_{30}+c_{14}c_{20}c_{31}+c_{14}c_{21}c_{30}-\\ c_{30}c_{40}c_{51}-c_{30}c_{41}c_{50}-c_{31}c_{40}c_{50}-2c_{12}c_{60}c_{61}-c_{13}c_{60}^{2} \end{array}$$

(A7) $$\begin{array}{l} h_{6}=c_{12}c_{22}c_{32}+c_{13}c_{21}c_{32}+c_{13}c_{22}c_{31}+c_{14}c_{20}c_{32}+c_{14}c_{22}c_{30}-c_{30}c_{41}c_{51}-c_{31}c_{40}c_{51}-\\ c_{31}c_{41}c_{50}-c_{32}c_{40}c_{50}-c_{12}c_{61}^{2}-2c_{13}c_{60}c_{61}-c_{14}c_{60}^{2} \end{array}$$

(A8) $$h_{7}=c_{13}c_{22}c_{32}+c_{14}c_{21}c_{32}+c_{14}c_{22}c_{31}-c_{31}c_{41}c_{51}-c_{32}c_{40}c_{51}-c_{32}c_{41}c_{50}-c_{13}c_{61}^{2}-2c_{14}c_{60}c_{61}$$

(A9) $$h_{8}=c_{14}c_{22}c_{32}-c_{32}c_{41}c_{51}-c_{14}c_{61}^{2}$$

where

(A10) $$\begin{array}{l} c_{10}=1\\ c_{11}=0\\ c_{12}=4(\eta_{N}+\alpha^{2})\\ c_{13}=-8{\alpha (\eta }_{P}+\bar{q})\\ c_{14}=4{\bar{q}}^{2}+8\eta_{P}\bar{q}+4\kappa_{p} \end{array}$$

(A11) $$\begin{array}{l} c_{20}=\mu_{s}\alpha^{2}\\ c_{21}=-2\alpha (\mu_{s}\bar{q}+\eta_{p}+\eta_{b})\\ c_{22}=\mu_{s}{\bar{q}}^{2}+2(\eta_{p}+\eta_{b})\bar{q}+\kappa_{p}+\kappa_{b} \end{array}$$

(A12) $$\begin{array}{l} c_{30}=\mu_{b}\alpha^{2}-\eta_{s}\\ c_{31}=-2\alpha (\mu_{b}\bar{q}+\eta_{b}+\eta_{f})\\ c_{32}=\mu_{b}{\bar{q}}^{2}+2(\eta_{b}+\eta_{f})\bar{q}+\kappa_{b}+1 \end{array}$$

(A13) $$\begin{array}{l} c_{40}=8\eta_{p}\alpha \\ c_{41}=-4(2\eta_{p}\bar{q}+\kappa_{p}) \end{array}$$

(A14) $$\begin{array}{l} c_{50}=2{\alpha \eta }_{p}\\ c_{51}=-(2\eta_{p}\bar{q}+\kappa_{p}) \end{array}$$

(A15) $$\begin{array}{l} c_{60}=2{\alpha \eta }_{b}\\ c_{61}=-(2\eta_{b}\bar{q}+\kappa_{b}). \end{array}$$
